# The inconsistent pathogenesis of endometriosis and adenomyosis: insights from endometrial metabolome and microbiome

**DOI:** 10.1128/msystems.00202-25

**Published:** 2025-04-22

**Authors:** Chao Li, Xinxin Xu, Xiaojie Zhao, Bin Du

**Affiliations:** 1 Department of Pathology, Shanghai First Maternity and Infant Hospital, School of Medicine, Tongji University 12476, Shanghai, China; 2 Shanghai Key Laboratory of Maternal Fetal Medicine, Shanghai Institute of Maternal-Fetal Medicine and Gynecologic Oncology, Clinical and Translational Research Center, Shanghai First Maternity and Infant Hospital, School of Medicine, Tongji University 12476, Shanghai, China; 3 Department of Gynecology, Shanghai First Maternity and Infant Hospital, School of Medicine, Tongji University 12476, Shanghai, China; San Diego State University, San Diego, California, USA

**Keywords:** endometriosis, adenomyosis, metabolome, microbiome, pathogenesis

## Abstract

Endometriosis (EM) and adenomyosis (AM) are interrelated gynecological disorders characterized by the aberrant presence of endometrial tissue and are frequently linked with chronic pelvic pain and infertility, yet their pathogenetic mechanisms remain largely unclear. In this cross-sectional study, we analyzed endometrial samples from 244 participants, split into 91 EM patients, 56 AM patients, and 97 healthy controls (HC). We conducted untargeted liquid chromatography-mass spectrometry (LC-MS) and 5R 16S rRNA sequencing to examine endometrial metabolome and microbiome profiles. Additionally, we integrated transcriptomic analysis using nine transcriptomic data sets to investigate the biological basis of these conditions. Metabolomic profiling and 16S rRNA sequencing revealed distinct metabolic and microbial signatures. Specific pathways, including linoleic acid and glycerophospholipid metabolism, show significant alterations in both conditions. Notably, four metabolites, including phosphatidylcholine 40:8 [PC(40:8)], exhibited marked changes in both EM and AM, suggesting shared pathological features. Furthermore, taxonomic analysis identified unique bacterial species associated with each condition, particularly those belonging to the phylum Proteobacteria, which correlated with altered metabolic signatures. Machine learning models demonstrated high predictive accuracy for differentiating between AM, EM, and HC based on metabolic and microbial signatures. Integrative analysis with transcriptomic data highlighted distinct pathways related to immune response and signaling transduction for each condition. Our study provides fresh insights into the pathogenesis of AM and EM through a multi-omic approach, suggesting potential inconsistencies in the underlying pathogenetic mechanisms.

**IMPORTANCE:**

Existing research highlighted a connection between endometriosis (EM) and adenomyosis (AM), underscoring their overlapping symptoms and potential shared pathophysiological mechanisms. Although the role of microbiota in inflammatory conditions has been acknowledged, comprehensive investigations into the endometrial microbiota in cases of EM and AM have been limited. Previous studies identified distinct microbial communities associated with these conditions; however, they were constrained by small sample sizes and a lack of integrated analyses of microbiota and metabolomics. Furthermore, the ongoing debate over whether EM and AM should be classified as separate diseases or related phenotypes emphasizes the necessity for further exploration of their molecular interactions. Our study uncovers distinct microbial and metabolic signatures associated with each condition, revealing both shared and unique pathways that may contribute to their pathogenesis. Furthermore, the integration of transcriptomic data offers valuable insights into the complex interactions underlying these disorders.

## INTRODUCTION

Endometriosis (EM) is a common chronic inflammatory condition characterized by dysmenorrhea, ectopic implantation of endometrial-like tissue, chronic pelvic pain, and infertility (1, 2). Notably, this condition affects only 10% of reproductive-age women, despite nearly all women experiencing some degree of retrograde menstruation, suggesting that its pathogenesis is multifactorial (3). Various immunological, neurological, and hormonal factors have been implicated in the etiology of EM and its associated symptoms (4). To date, no singular theory has adequately explained the wide range of clinical manifestations and pathological features of EM.

Adenomyosis (AM) is another prevalent gynecological disorder characterized by the presence of endometrial glands and stroma embedded within the myometrium. It typically presents with pelvic pain, abnormal uterine bleeding, and infertility (5, 6). Recent research supports the hypothesis that AM arises from the disruption of the normal endometrial-myometrial junction, leading to the invagination of the endometrial basal layer into the myometrium (7, 8). EM and AM are considered closely related conditions, with women diagnosed with EM exhibiting a higher incidence of AM and irregular junctional zones (9). Prior studies suggest that extrinsic AM may originate from pelvic EM (10), and focal AM in the outer myometrium may be linked to deep infiltrating EM lesions (11). However, uncertainty persists regarding the specific etiologies of EM and AM, and the ongoing debate as to whether they represent distinct phenotypes of a single disease continues (12, 13).

Emerging evidence suggests that microbiota function as an additional organ, actively participating in the maintenance of human physiology (14). Dysbiosis, or microbial imbalance, has been confirmed as a pathological state (15), contributing to a pro-inflammatory environment (16) and influencing estrogen levels (14, 17, 18). While differential microbiota and alterations in microbial architecture have been documented in the gut, vagina, and peritoneal fluid of patients with EM (19) and AM (20–22), research on the endometrial microbiota—whose communities differ from those found in other sites (23, 24)—in these conditions remains largely unexplored. Several studies have shown that the endometrial microbiota in individuals with EM (23, 25, 26) and AM (21, 23, 27) is colonized by bacterial species distinct from those in healthy women. However, most of these investigations have been conducted on small sample sizes, and comprehensive analyses combining the endometrial microbiota of both EM and AM patients have yet to be published.

One potential mechanism through which the microbiota influences host physiology is by producing specific metabolites. Altered levels of certain metabolites have been particularly associated with immune response, cell proliferation, and cell migration in EM and AM (28–33). To date, only a limited number of studies have concurrently evaluated the microbiota and metabolomics from the same subjects, thus elucidating the interplay between microbiota and metabolites in the context of EM (33–35). Nevertheless, there is a lack of data on the simultaneous analysis of the endometrial microbiome and metabolome to fully understand how these factors interact with the host in both diseases, as well as whether specific microbes and metabolites collaboratively influence the onset of EM and AM.

In this study, we conducted an integrated analysis of the endometrial metabolome through untargeted liquid chromatography-mass spectrometry (LC-MS) metabolomic profiling, alongside the intra-endometrial microbiota profile utilizing 5R 16S rRNA sequencing (36) in patients with EM and AM, and compared these profiles to those of healthy subjects. Additionally, we performed a meta-analysis of nine transcriptomic data sets obtained from NCBI to identify differentially expressed genes involved in the pathogenesis of EM and AM. The aim of our investigation is to provide additional evidence to determine whether EM and AM represent two distinct entities or are simply different phenotypes of a single disease, based on their metabolomic, microbiomic, and transcriptomic profiles.

## RESULTS

### Description of the study population

**Fig 1:**
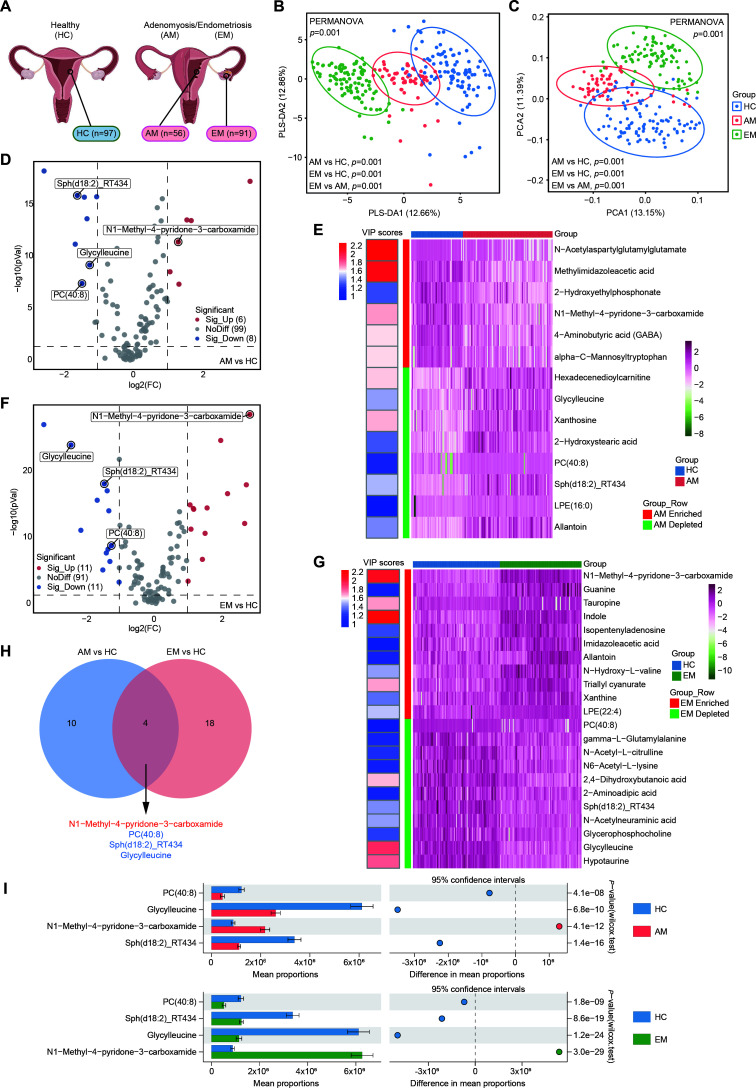
Metabolic profiling analysis of the endometrial metabolome among EM, AM, and HC groups. (**A**) Schematic representation of the collected tissue biopsy samples. HC specimens were obtained from the eutopic endometrium of individuals without endometriosis or adenomyosis. EM and AM samples were collected from individuals diagnosed with EM and AM, respectively. (**B**) PLS-DA was performed for the EM, AM, and HC groups. *P* values were calculated using the PERMANOVA method and Bray–Curtis distance. (**C**) PCA was conducted for the EM, AM, and HC groups. *P* values were computed using the PERMANOVA method and Bray–Curtis distance. (**D and F**) Volcano plots depicting the metabolite changes between AM versus HC (**D**) and EM versus HC (**F**) are presented. Each dot represents a metabolite identified in the samples; blue dots indicate downregulated metabolites, while red dots indicate upregulated metabolites. Significantly altered metabolites were determined using the VIP score from pairwise PLS-DA analysis and the Mann–Whitney *U* test, with significance thresholds set at VIP > 1, adjusted *P* < 0.05, and |log_2_FC| ≥ 1. The marked metabolites identify those that are significantly altered and shared between the AM and EM groups. (**E and G**) Heatmaps display the significantly altered metabolites between AM and HC (**E**) and between EM and HC (**G**). (**H**) A Venn diagram illustrates the shared significantly altered metabolites between the AM and EM groups. (**I**) The Mann–Whitney *U* test was performed to analyze between-group differences in the shared significantly altered metabolites between AM and HC or EM and HC groups.

Our study included 244 subjects, namely, 91 diagnosed with EM, 56 with AM, and 97 healthy controls (HC) (Fig. 1A). All diagnoses of EM and AM were made based on surgical findings, which were later validated by pathologists. Demographic information and clinical characteristics for each participant are summarized in Table 1. The subjects were matched for age, body mass index (BMI), menstrual cycle phase, and parity (*P* > 0.05). In terms of clinical symptoms, patients with EM and AM did not demonstrate significantly higher levels of dysmenorrhea compared to the HC. Notably, patients with elevated serum albumin levels and reduced levels of alanine aminotransferase, aspartate aminotransferase, and triglycerides were found to have a higher risk of developing EM (*P* < 0.05). Additionally, the levels of CA125, previously investigated as potential biomarkers for adenomyosis (37–39), endometriosis (40), as well as endometrial and ovarian cancers (41, 42), did not show significant differences among the endometrial samples in our cohort. Detailed baseline characteristics of the subjects are provided in Table S1.

**TABLE 1:** Baseline clinical characteristics of the women included in the study^*a*^

Parameter	HC	AM	EM	*P* value	
	(*n* = 97)	(*n* = 56)	(*n* = 91)	AM vs HC	EM vs HC
Age (years)	41 ± 3.72	40 ± 4.56	39 ± 5.88	0.23	0.10
BMI (kg/m^2^)	23.28 ± 2.93	22.81 ± 2.68	22.60 ± 2.88	0.33	0.11
Menstrual cycle phase, *N* (%)				0.89	0.67
Proliferative	92 (94.8)	52 (92.6)	85 (93.4)		
Secretory	5 (5.2)	4 (7.4)	6 (6.6)		
Dysmenorrhea, *N* (%)				0.12	0.42
Yes	51 (52.6)	37 (66.1)	44 (48.4)		
No	46 (47.4)	19 (33.9)	47 (51.6)		
Parity, *N* (%)				0.53	0.53
Yes	95 (97.9)	53 (94.6)	82 (90.1)		
No	2 (2.1)	3 (5.4)	9 (9.9)		
Hormonal treatment, *N* (%)				0.74	0.23
Yes	14 (14.4)	7 (12.5)	8 (8.8)		
No	83 (85.6)	49 (87.5)	83 (91.2)		
D-dimer (<0.55 mg/L)	0.40 ± 0.41	0.34 ± 0.30	0.3 ± 0.31	0.36	0.06
CA125 (0–35 U/mL)	67.71 ± 79.63	80.11 ± 72.85	61.75 ± 44.56	0.34	0.53
Alanine aminotransferase (1–40 U/L)	17.23 ± 11.30	17.54 ± 11.01	14.22 ± 8.31	0.87	**0.04**
Aspartate aminotransferase (2–40 U/L)	19.76 ± 5.70	20.45 ± 5.85	17.91 ± 4.53	0.48	**0.02**
Albumin (33–55 g/L)	42.66 ± 2.71	43.35 ± 2.59	44.65 ± 2.88	0.13	**0.00**
Prealbumin (180–390 mg/L)	229.04 ± 37.69	233.32 ± 45.41	227.77 ± 33.84	0.53	0.81
Urea (2.5–6.5 mmol/L)	4.87 ± 1.19	5.17 ± 1.27	4.73 ± 1.46	0.15	0.48
Creatinine (53–115 umol/L)	49.93 ± 12.17	49.80 ± 13.92	49.23 ± 12.13	0.95	0.69
Serum glucose (3.9–6.1 mmol/L)	5.13 ± 0.87	5.33 ± 1.08	4.91 ± 0.70	0.22	0.07
Cholesterol (2.8–5.85 mmol/L)	4.73 ± 0.81	4.74 ± 0.86	4.78 ± 0.80	0.94	0.63
Triglyceride (0.34–1.8 mmol/L)	1.39 ± 0.91	1.40 ± 1.02	0.97 ± 0.60	0.95	**0.00**
High-density lipoprotein (1–1.56 mmol/L)	1.45 ± 0.78	1.51 ± 1.00	1.53 ± 0.35	0.71	0.38
Low-density lipoprotein (1.6–3.36 mmol/L)	2.93 ± 0.77	2.94 ± 0.86	2.90 ± 0.79	0.94	0.78

^*a*^ Data are expressed as the number or mean ± SD. BMI, body mass index; HC, healthy control; AM, adenomyosis; EM, endometriosis. *P* values for categorical and continuous variables were obtained from the chi-square test and *t*-test, respectively. Bold values in column "EM vs HC" indicates *P* < 0.05.

### Metabolomic profiling reveals a disparity among AM, EM, and HC

A total of 515 metabolites were quantified from endometrial tissue samples. The distribution of quality control (QC) samples appears to show a generally consistent pattern, potentially suggesting acceptable instrument stability (Fig. S1 and Table S2). The metabolomic profiles of these samples exhibited distinct clustering for the EM, AM, and HC groups, as demonstrated in both partial least squares discriminant analysis (PLS-DA; permutational multivariate analysis of variance [PERMANOVA], *P* = 0.001; Fig. 1B) and principal component analysis (PCA; PERMANOVA, *P* = 0.001; Fig. 1C). This clustering suggests notable shifts in endometrial metabolites associated with AM and EM. To identify specific metabolites significantly altered in relation to AM and EM, pairwise comparisons between groups were performed. In the comparison of AM to HC, 14 metabolites showed significant alterations, with six metabolites being enriched and eight depleted (variable importance in projection [VIP] > 1, adjusted *P* < 0.05, and fold change [FC] > 2; Fig. 1D and E). Similarly, in the EM versus HC comparison, 22 metabolites exhibited differential abundance, comprising 11 enriched and 11 depleted metabolites (VIP > 1, adjusted *P* < 0.05, and FC > 2; Fig. 1F and G). Notably, four metabolites, including N1-methyl-4-pyridone-3-carboxamide, phosphatidylcholine 40:8 [PC(40:8)], Sph(d18:2)_RT434, and glycylleucine, were consistently altered, either enriched or depleted, in both AM and EM compared to HC (Fig. 1H and I). These findings highlight significant variability in the metabolic community structure and the specific metabolites present in the contexts of AM and EM.

### MetOrigin tracing analysis of differential metabolites

**Fig 2:**
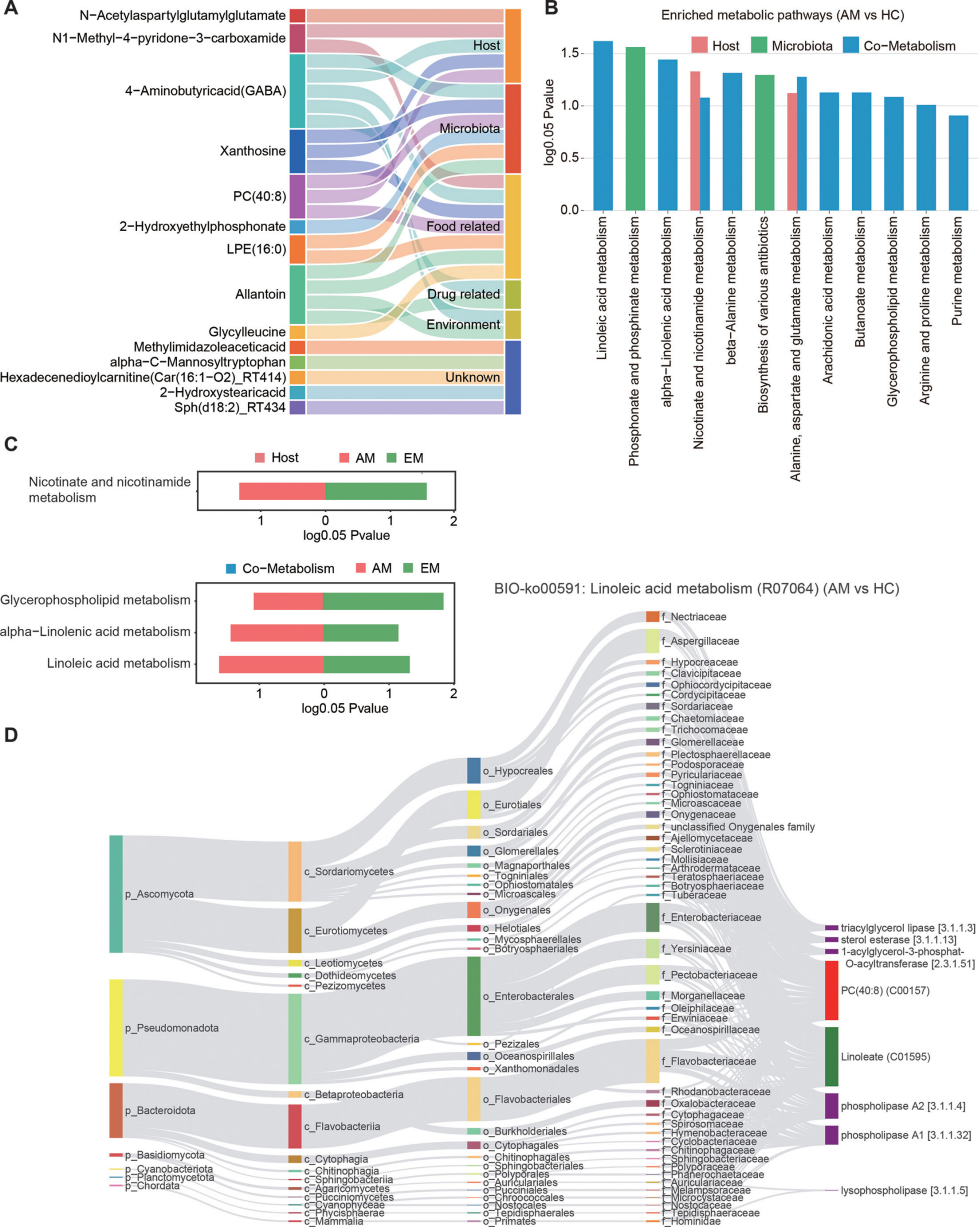
MetOrigin metabolite traceability analysis and Sankey diagram of differential metabolites. (**A**) Sankey diagram illustrates the origins of differential metabolites. Among these, only one out of six microbial-associated metabolites and one out of five host-associated metabolites was exclusively originated from microbial or host sources, respectively, suggesting a degree of uncertainty in the annotation of these metabolites. (**B**) Histogram representing the enrichment analysis of differential metabolic pathways. (**C**) Histogram displaying the shared host and bacteria–host cometabolic pathways in AM and EM interactions. (**D**) Sankey diagram illustrating the MetOrigin analysis of linoleic acid metabolism. Dark red bars, metabolic substrates; dark green bars, metabolic products; purple bars, metabolic enzymes.

To investigate the enrichment and pathways of the differential metabolites, we conducted metabolite traceability and source-based metabolic function analyses. By comparing the AM group with the HC group, we identified differential metabolites associated with AM. Specifically, the microbial-associated cluster contained six metabolites (one exclusively microbial and others potentially originated from host, food, drugs, or environmental factors), while the host-associated cluster comprised five metabolites (one uniquely host-originated and others sourced from microbiota, food, drugs, or environment) (Fig. 2A and Table S3). The origin-based functional analysis revealed 11 significantly modulated pathways (*P* < 0.05), with nine of these—excluding phosphonate and phosphinate metabolism and beta-alanine metabolism—being associated with inflammatory responses. Notably, linoleic acid metabolism showed the most significant modulation (Fig. 2B). In the comparison between the EM group and the HC group, we identified 13 microbial-associated metabolites originating from multiple sources (including host, microbiota, food, drugs, or environment), and nine host-associated metabolites that also derive from various sources (including host, microbiota, food, or drugs) (Fig. S2A and Table S3). The origin-based functional analysis for this comparison highlighted 13 significantly modulated pathways related to inflammation (*P* < 0.05), with glycerophospholipid metabolism emerging as the most critical factor (Fig. S2B). Remarkably, four metabolic pathways, including one host-specific and three bacteria–host pathways, were found to be significantly altered in both the AM and EM groups (Fig. S2C; Fig. 2C).

To visually depict the biological and statistical correlations between microbiota and metabolites, we constructed Bio-Sankey networks using MetOrigin analysis. In the context of linoleic acid metabolism, PC(40:8) was identified as a significant metabolite, with the phylum Proteobacteria playing a crucial role in the metabolic reaction R07064 within this pathway (Fig. 2D). Additionally, PC(40:8) serves as a key metabolite in glycerophospholipid metabolism (R01315), predominantly produced through the actions of phospholipase A1 and phospholipase A2, resulting in the formation of 1-acyl-sn-glycero-3-phosphocholine (Fig. S2D) and linoleate (Fig. 2D) for EM and AM, respectively. Notably, PC(40:8) is implicated in both linoleic acid and glycerophospholipid metabolisms, with Proteobacteria identified as the primary phylum associated with these processes (Fig. 2; Fig. S2). Various choline-utilizing microbiota possess the ability to metabolize choline into either phosphatidylcholine (PC) or lysophosphatidylcholine (lysoPC) (43), a process that may modulate phospholipase A2 activity, which has been linked to both EM (29, 44–46) and AM (29). Consequently, assessing the microbiome is critical for identifying key microbial species that mediate metabolic changes influencing the development of AM and EM.

### Taxonomic characterization of the microbiota among AM, EM, and HC

**Fig 3:**
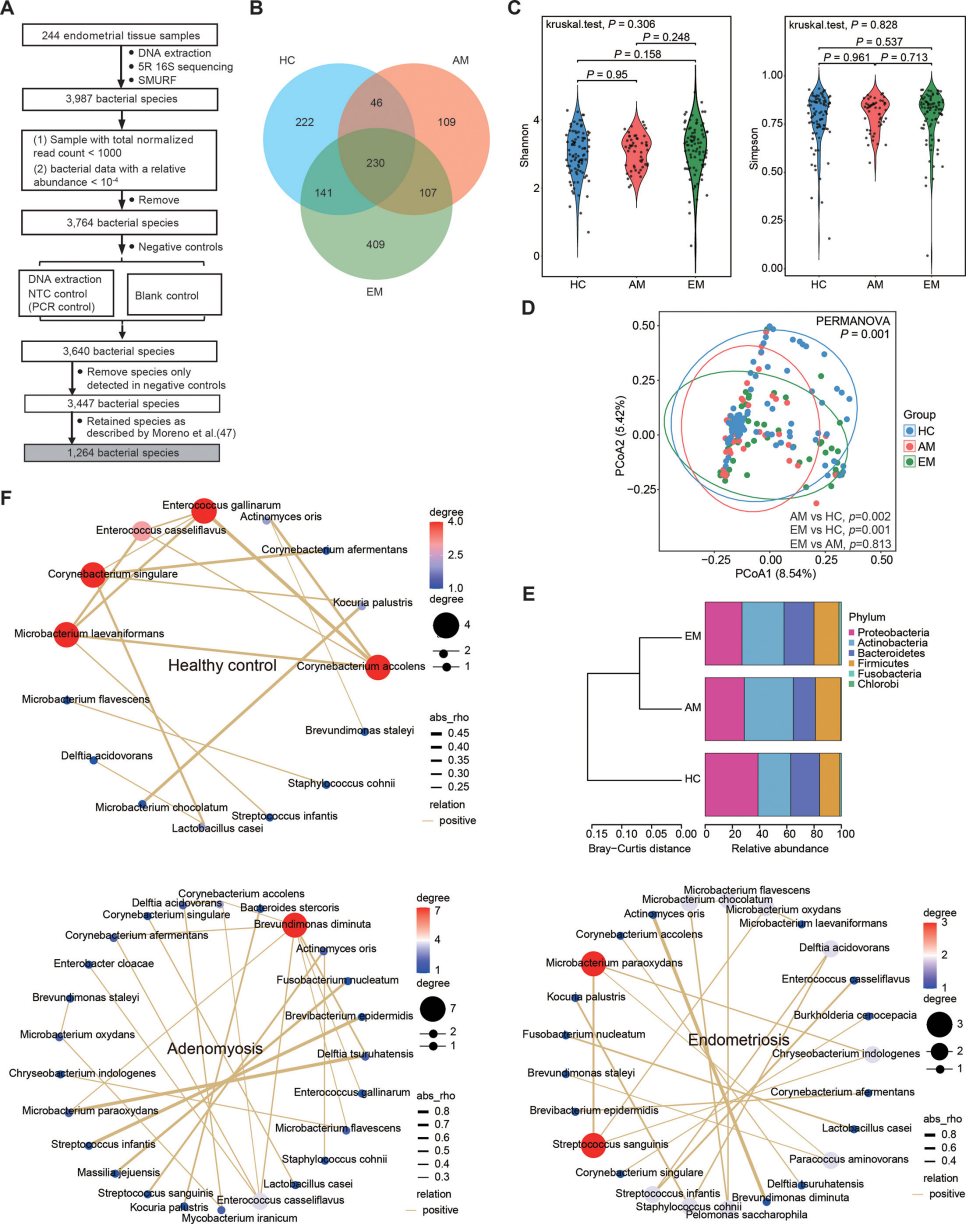
Analysis of endometrial microbiota diversity and structure. (**A**) Contamination removal procedure for 5R 16S rRNA sequencing. NTC, no template control. (**B**) A Venn diagram illustrating the observed bacterial species among EM, AM, and HC individuals. (**C**) Alpha diversity of the microbiota presented as estimated by the Shannon and Simpson indices. *P* value was calculated using the Kruskal–Wallis rank-sum test among three groups and the Mann–Whitney *U* test for comparisons between two groups. (**D**) Beta diversity evaluated by PCoA based on Bray–Curtis distance. *P* value was determined using the PERMANOVA test for comparison among the three groups and the Mann–Whitney *U* test for two-group analyses. (**E**) Hierarchical clustering based on Bray–Curtis distances utilizing the average relative proportions of the top 10 abundant phyla across the three groups. (**F**) Pearson’s correlation analysis was conducted to assess the differences in the abundance of the top 30 species across the three groups. Only correlations with a *P* value < 0.05 are presented. The size of the nodes represents the relative abundance of each species, while the color gradient of the connecting lines indicates the *R* value, with positive correlations depicted in orange.

**Fig 4:**
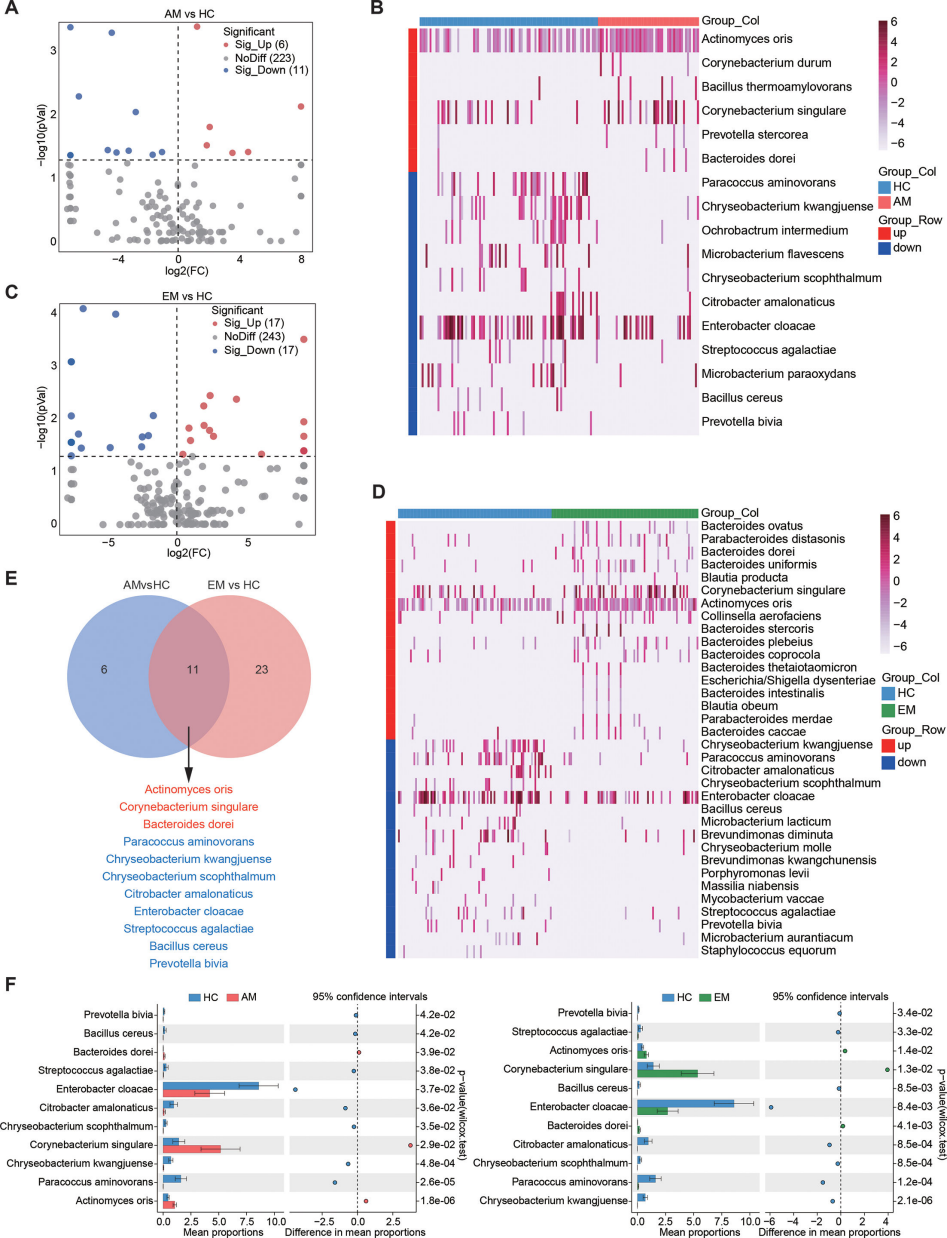
Differential analysis of endometrial microbiota at the species level. (**A and C**) Volcano plots depicting differential bacterial abundance in the AM group (**A**) and the EM group (**C**) compared to the HC group. Each blue dot represents a bacterium that is downregulated, whereas each red dot represents a bacterium that is upregulated. Significantly altered bacterial abundances were determined using the Mann–Whitney *U* test, with a significance cutoff set at *P* < 0.05. The black circles highlight the differentially abundant bacteria that are shared between the AM and EM groups. (**B and D**) Heatmaps illustrating the differentially abundant bacterial species between the AM and HC groups (**B**) and between the EM and HC groups (**D**). (**E**) A Venn diagram illustrating the differentially abundant bacterial species that are shared between the AM and EM groups. (**F**) The Mann–Whitney *U* test was conducted to analyze between-group differences in the shared differentially abundant bacterial species between the AM and HC groups or the EM and HC groups.

The amplicon-based 5R 16S rRNA gene sequencing technique (36) was employed to examine the taxonomic composition of the endometrial tissues. A total of 1,264 bacterial species were detected in the endometrial samples, utilizing a stringent set of filters to minimize contamination, as outlined previously (36, 47) (Fig. 3A and Tables S4–S6). Among these bacterial species, 222 (17.6%) were unique to the HC group, while the AM and EM groups had 109 (8.6%) and 409 (32.4%) unique bacterial species, respectively (Fig. 3B). Alpha diversity analysis revealed no significant differences in the Shannon and Simpson indices between the AM and EM groups when compared to the HC group (Fig. 3C). In contrast, principal coordinates analysis (PCoA) using Bray–Curtis distance illustrated significant differences between the AM and EM groups relative to the HC group (PERMANOVA, *P* = 0.001) (Fig. 3D). The classification of bacterial species indicated that the distribution of dominant bacteria in the AM and EM groups was notably less similar to the HC group at the phylum, genus, and species levels (Fig. 3E; Fig. S3). Furthermore, the occurrence network of bacterial species exhibited a modest increase in complexity from the HC group to the AM and EM groups, suggesting a strengthening of connections among bacterial species, likely due to the enhanced presence of specific microbial species (Fig. 3F). Further analysis utilizing volcano plots revealed 17 differentially abundant bacterial species (DABs; *P* < 0.05) in the AM group compared to the HC group, which included 6 enriched and 11 depleted bacterial species (Fig. 4A and B). Additionally, a total of 34 DABs (*P* < 0.05; 17 enriched and 17 depleted) were identified between the EM and HC groups (Fig. 4C and D). Notably, among these species, 11 exhibited a consistent trend when both the AM and EM groups were compared to the HC group (Fig. 4E and F). These findings suggest a complex relationship in the abundance of endometrial microbiota between AM and EM.

To further explore the potential associations between the endometrial microbiome and the metabolome, we conducted a Pearson’s correlation analysis. This analysis evaluated all altered microbial species alongside differential metabolites in the AM and HC groups, as well as in the EM and HC groups. As shown in Figure S4A, the abundances of *Chryseobacterium kwangjuense*, *Bacillus cereus*, and *Enterobacter cloacae* exhibited significant positive correlations with the levels of PC(40:8) when comparing the AM and HC groups. Similarly, in the EM group relative to the HC group, *C. kwangjuense*, *Microbacterium aurantiacum*, *B. cereus*, and *E. cloacae* showed the strongest associations with PC(40:8), with three of these species also present in the AM group (Fig. S4B). Notably, one of these bacterial species, including *E. cloacae*, belongs to the phylum Proteobacteria. This finding corresponds with the results of the MetOrigin metabolite tracing analysis (Fig. 2; Fig. S2), suggesting that Proteobacteria may influence changes in PC levels within the linoleic acid and glycerophospholipid metabolic pathways. The observation indicates a shared modulation of microbiota and metabolites in the development of both AM and EM, emphasizing that various microbial species often exhibit similar metabolic capacities that extend beyond mere microbiota composition (48).

### Dissimilar correlations of AM-/EM-related metabolites and microbiota with clinical indices

**Fig 5:**
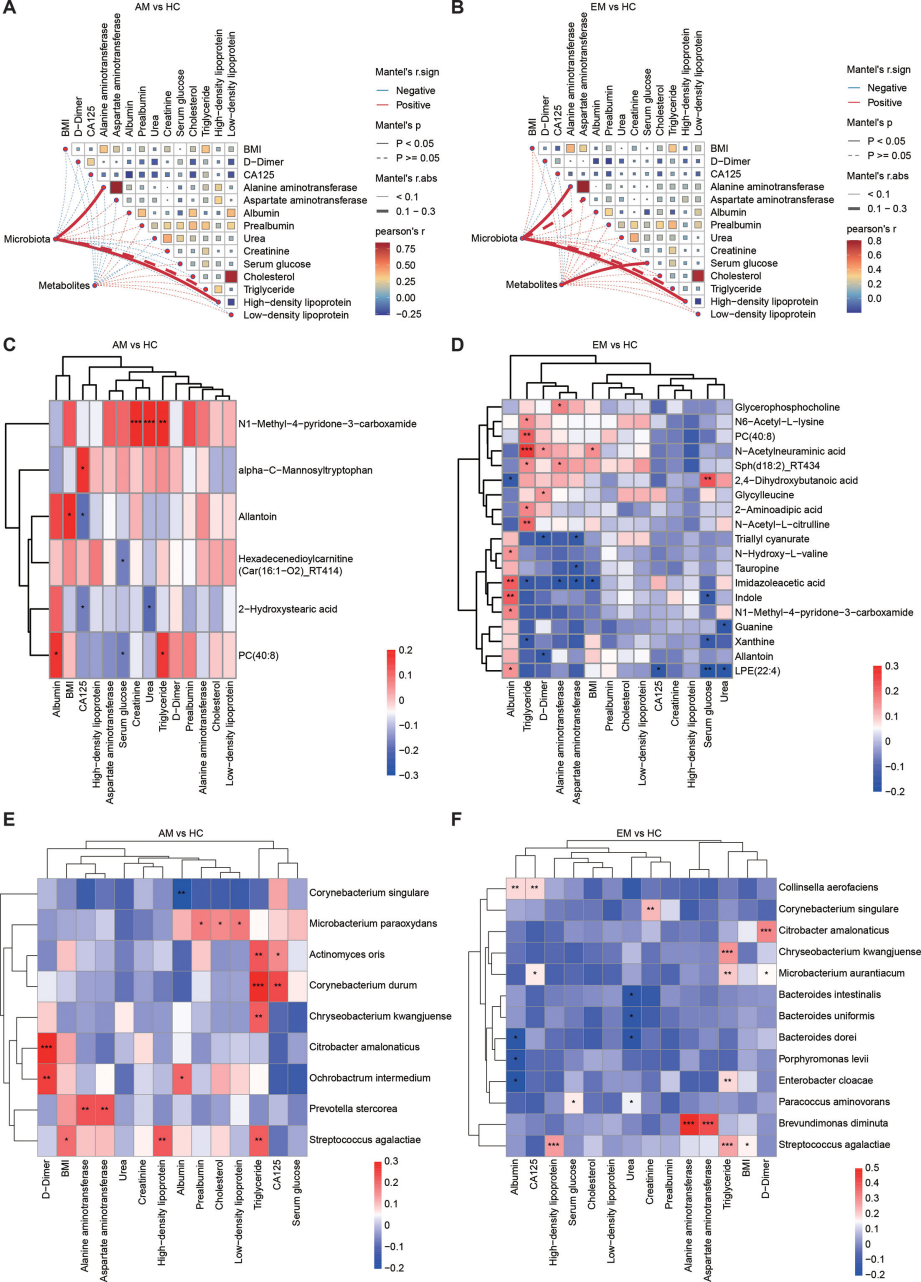
Correlation analysis of altered endometrial metabolites and microbial species with clinical indices. (A and B) Correlation analysis between 14 clinical indices and the overall endometrial metabolic and microbial communities in patients with AM and HC (**A**), as well as from patients with EM and HC (**B**). (C and D) Pearson’s rank correlation to assess the relationships between differentially abundant metabolites and 14 clinical indices derived from AM patients and HC (**C**), as well as from EM patients and HC (**D**). The analysis includes only those metabolites that demonstrated a correlation with at least one clinical index at a significance level of *P* < 0.05 (*), *P* < 0.01 (**), or *P* < 0.001 (***). (E and F) Pearson’s rank correlation to assess the relationships between differentially abundant species and 14 clinical indices derived from AM patients and HC (**C**), as well as from EM patients and HC (**D**). The analysis includes only those species that demonstrated a correlation with at least one clinical index at a significance level of *P* < 0.05 (*), *P* < 0.01 (**), or *P* < 0.001 (***).

To further explore potential correlations between changes in endometrial metabolites and microbiota with clinical indices, we performed Pearson’s correlation analysis. The results suggested significant correlations in both the AM and EM groups (*P* < 0.05), which were validated by the Mantel test (Fig. 5A and B). Notably, significant correlations were found between the microbial community and alanine aminotransferase (*r* = 0.18 for AM; *r* = 0.20 for EM) and high-density lipoprotein in both groups (*r* = 0.19 for AM; *r* = 0.23 for EM), as well as between the metabolic community and serum glucose (*r* = 0.16), although this latter association was observed exclusively in the EM group.

Subsequent analysis of individual features revealed specific associations. In patients with AM, albumin showed a positive correlation with the metabolite PC(40:8) and the bacterial species *Ochrobactrum intermedium*. Similarly, CA125 exhibited the strongest positive correlation with alpha-C-mannosyltryptophan and *Corynebacterium durum*, while BMI was positively correlated with allantoin and *Streptococcus agalactiae* (Fig. 5C and E). In contrast, among patients with EM, albumin was positively correlated with the metabolites such as imidazoleacetic acid and indole, as well as with the bacterial species *Collinsella aerofaciens*. Moreover, CA125 showed a negative correlation with LPE(22:4) and a positive correlation with *C. aerofaciens*, whereas BMI exhibited both positive and negative correlations with the metabolites and a negative correlation with *S. agalactiae* (Fig. 5D and F). Additional correlations with serum glucose, urea, alanine aminotransferase, aspartate aminotransferase, triglyceride, and D-dimer were also identified in the EM group. Notably, triglyceride levels were positively associated with the metabolite PC(40:8) and the bacterial species *C. kwangjuense* and *S. agalactiae* in both the AM and EM groups (Fig. 5C–F). All these relationships were further assessed through linear regression analysis (Fig. S5). Overall, these findings suggest that disturbances in metabolic and microbial communities may serve as potential biomarkers linking alterations in endometrial metabolites and microbiota to clinical indices.

### Multi-omics signatures-based prediction of AM and EM

**Fig 6:**
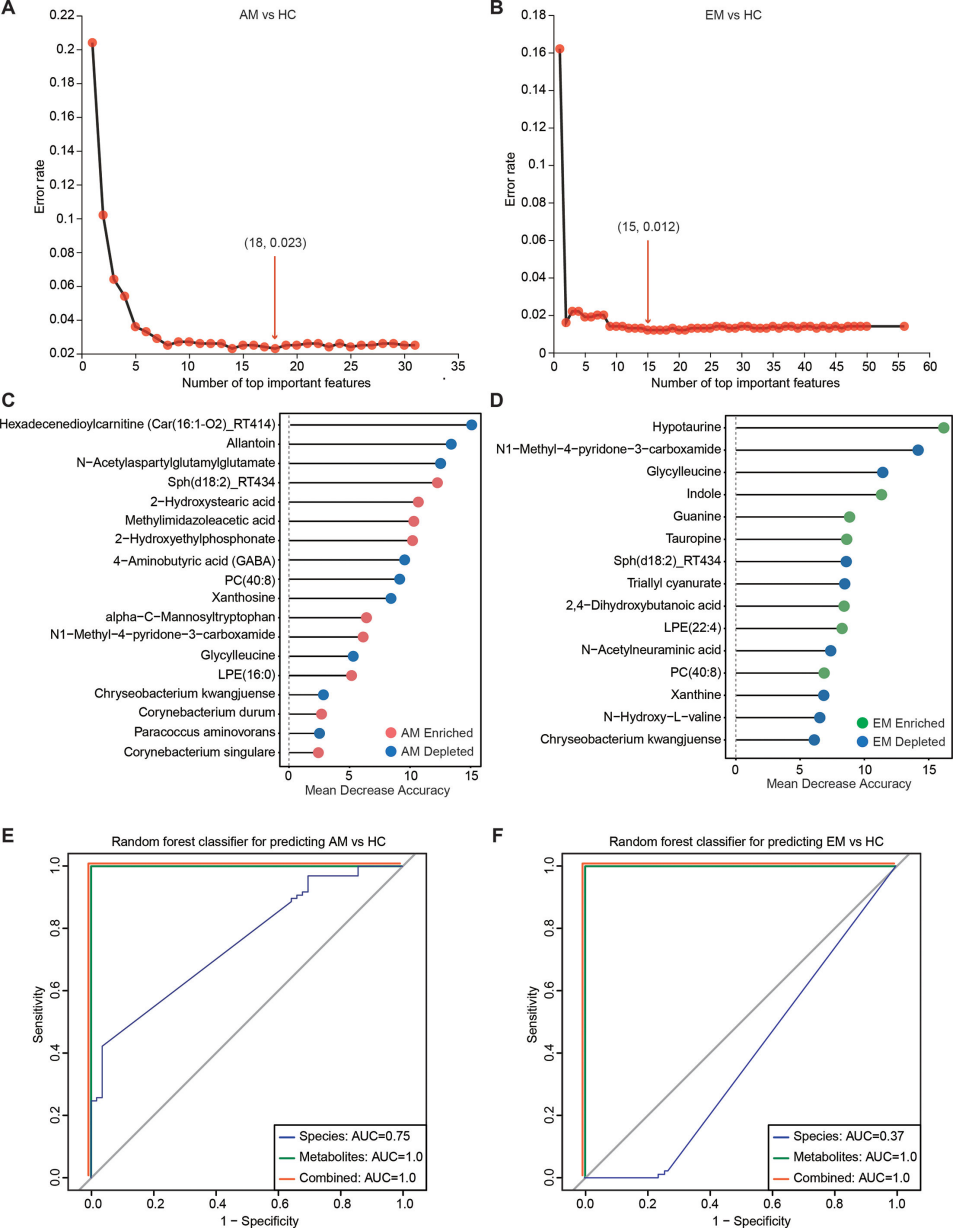
Multi-omics signatures-based prediction of AM and EM. (A and B) The cross-validation curves illustrate the results from 20 trials of 10-fold cross-validation process comparing the AM group with the HC group (**A**), and the EM group with the HC group (**B**), based on the differential metabolic and microbial features. The number of features corresponding to the minimum cross-validation error is highlighted in red font. (C and D) A total of 19 and 18 endometrial microbial and metabolic biomarkers, derived from individuals in the AM and HC groups (**C**), and the EM and HC groups (**D**), respectively, were identified as achieving the lowest classification error. These biomarkers were obtained using the mean decrease accuracy tool from the RF algorithm and were ranked according to their contributions to classification accuracy following permutation analysis. The color associated with each biomarker indicates its enrichment in the AM (red), EM (green), or HC (blue) participants. (E and F) The ROC curves of the RF model, utilizing discriminatory signatures (5 species and 14 metabolites for AM; 1 species and 17 metabolites for EM), are presented for the 153 samples in the AM cohort (**E**) and the 188 samples in the EM cohort (**F**).

To identify potential signatures that could serve as predictors of endometrial status, we developed random forest (RF) classifiers using 10-fold cross-validation and receiver operating characteristic (ROC) curves, based on differential microbial species and metabolic profiles. In patients with AM, we developed a panel of microbiota and metabolite signatures that included four microbial species and 14 metabolites (Fig. 6A and C). The identified disease signatures included microbial species specific to the AM condition, such as *C. kwangjuense*, *Paracoccus aminovorans*, and *C. durum*, alongside a unique cluster of metabolites. Among these, N-acetylaspartylglutamylglutamate, enriched in AM, emerged as the most distinctive signature associated with this condition. Similarly, we established a combinatorial marker panel for patients with EM, which included one microbial species and 14 metabolites, with indole identified as the most discriminatory metabolite prevalent in this condition (Fig. 6B and D).

We subsequently evaluated the potential utility of endometrial microbiota and metabolomic markers for identifying AM and EM. Notably, the predictive model indicated that metabolic signatures exhibited high sensitivity in detecting AM, achieving an area under the curve (AUC) value of 1.0 (Fig. 6E). The combination of microbial species and metabolites significantly improved classification accuracy compared to using microbial species alone (AUC = 1.0 versus 0.75). We also validated discrimination between the EM and HC groups (Fig. 6F), with the metabolic signatures demonstrating superior accuracy in distinguishing EM patients from HC, achieving an AUC of 1.0. When combined with microbial marker, the AUC also reached 1.0, greatly surpassing the performance of microbiota biomarker used independently (AUC = 0.37). To further distinguish between AM and EM, we employed RF modeling to analyze differential metabolites and microbiota (Fig. S5D-S6A), resulting in a panel of 15 metabolite signatures that successfully distinguished EM from AM, with an AUC of 1.0 (Fig. S6E). These findings underscore the potential for developing these microbial and metabolic classifiers as promising tools for identifying populations with AM and EM.

### Integrated analysis of patient metabolomics with transcriptomics data reveals valuable metabolites and genes related to AM and EM

**Fig 7:**
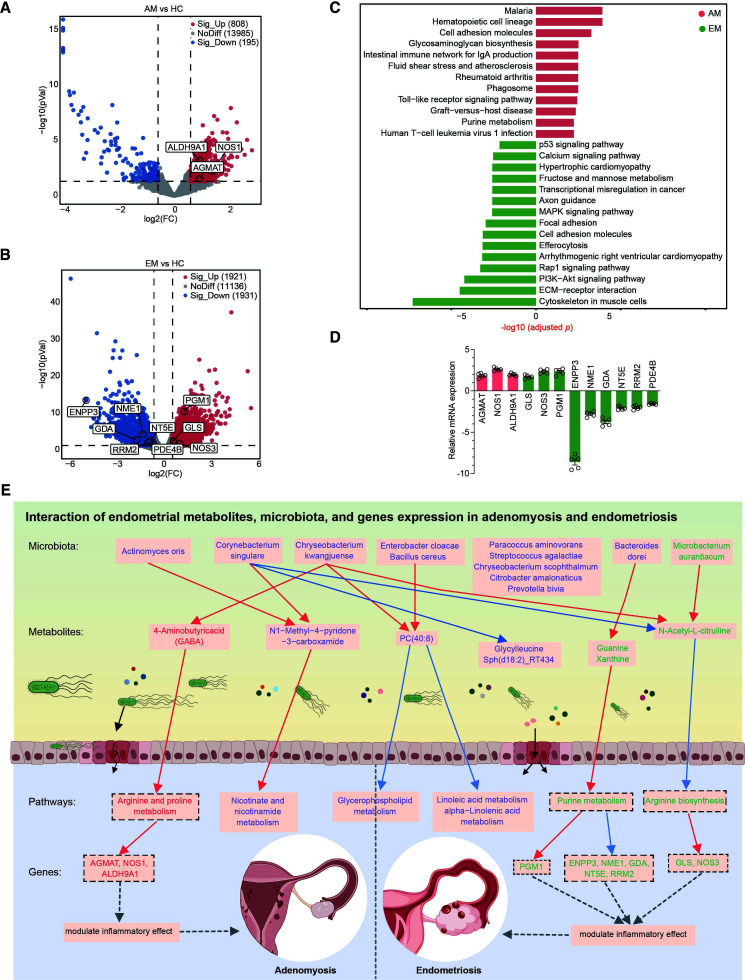
Integrated analysis of patient metabolomics with transcriptomics data. (A and B) Volcano plots depicting differentially expressed genes in the AM and EM groups compared to the HC group. Blue dots represent downregulated genes, while red dots represent upregulated genes. Significantly altered genes were identified using DESeq2, with a threshold of |log_2_FC| ≥ 0.585 and a *P* value < 0.05. The highlighted genes are indicative of their association with disease occurrence. (**C**) A bar plot displaying the 15 top enriched pathways, ordered by -log_10_(*P*), for the AM group (red) and the EM group (green) in the data set. (**D**) qRT-PCR verification of transcriptomic data related to panels A and B. (**E**) Schematic representation of the interactions between endometrial metabolites, microbiota, and gene expression in adenomyosis and endometriosis. The color gradient ranges from blue, indicating a negative correlation, to red, signifying a positive correlation. The use of purple fonts denotes metabolites, microbiota, and pathways that are common to both AM and EM. In contrast, red fonts indicate elements unique to AM, while green fonts represent those exclusive to EM. The black dashed box indicates that the transcriptomic-metabolomic correlations should be regarded as putative (nominal *P* < 0.05, FDR-adjusted *P* > 0.05).

To investigate the biological basis for the disparities among AM, EM, and HC, we conducted a meta-analysis to elucidate their transcriptomic differences. Our analysis incorporated a total of 52 HC samples, 22 AM samples, and 19 EM samples obtained from nine publicly available data sets: GSE135485, GSE153739, GSE153740, GSE157718, GSE171653, GSE185392, GSE193928, GSE212787, and GSE228005. We identified 1,003 significant differentially expressed genes (DEGs) in the AM group, comprising 808 upregulated and 195 downregulated genes (Fig. 7A). In the EM group, we detected 3,852 significant DEGs, with 1,921 genes upregulated and 1,931 downregulated, using a threshold of |log_2_FC| ≥ 0.585 and *P* < 0.05 from an initial pool of 14,988 filtered genes (Fig. 7B). Subsequently, we employed the Kyoto Encyclopedia of Genes and Genomes (KEGG) to analyze enriched pathways in the AM and EM groups, identifying 12 and 24 KEGG pathways, respectively, at a significance level of adjusted *P* < 0.05 (Table S7). We ranked these enriched pathways according to their *P* values to extract the most significantly enriched biological processes for each group. Notably, pathways related to hematopoietic cell lineage, intestinal immune network for immunoglobulin A (IgA) production, and toll-like receptor signaling were found to be upregulated in the AM patients. Conversely, pathways associated with extracellular matrix–receptor interaction, PI3K-Akt signaling pathway, Rap1 signaling pathway, MAPK signaling pathway, and calcium signaling pathway were predominantly activated in the EM patients (Fig. 7C). These findings highlight the divergent biological characteristics of the two patient populations, revealing that the AM patients exhibit an exuberant immune response (49), while the EM patients demonstrate variability in signal transduction pathways.

To investigate potential transcriptome–metabolome interactions, we performed a joint KEGG pathway enrichment analysis. While no shared pathways emerged in either the AM or EM groups, we identified disease-relevant DEGs with putative functional connections to metabolic pathways. In the AM group, inflammatory process-related genes (AGMAT [50, 51], NOS1 [52], ALDH9A1 [53, 54]) showed associations with arginine and proline metabolism (nominal *P* < 0.05, false discovery rate [FDR]-adjusted *P* > 0.05; Table S8). Similarly, EM-specific DEGs involved in arginine biosynthesis (GLS [55], NOS3 [56]) and purine metabolism (ENPP3 [57], PGM1 [58], NME1 [59], GDA [60], NT5E [61], RRM2 [62], PDE4B [63]) displayed pathway-level correlations with metabolomic changes, although these associations also remained significant only at the nominal *P* value level (Table S8). To validate these findings, we conducted a quantitative reverse transcription polymerase chain reaction (qRT-PCR) on the above 12 DEGs sharing KEGG pathways with metabolome data. Their expression patterns consistently matched RNA-seq results (Fig. 7D and Table S9), strengthening the biological relevance of these nominal associations. Collectively, integrative analysis revealed substantial disparities between the AM and EM patients, manifesting as distinct transcriptional profiles coupled with metabolic dysregulation. While pathway-level overlaps lacked adjusted statistical significance, gene-specific associations at nominal thresholds highlight biologically plausible interactions warranting mechanistic investigation.

## DISCUSSION

EM and AM are two interrelated gynecological conditions characterized by the ectopic implantation and proliferation of endometrial tissue. Previous research suggests that these disorders may share a common pathophysiology, as evidenced by similarities in cellular composition and resistance to progestogens within their respective lesions (13, 49); however, the underlying mechanisms remain inadequately understood. In this study, we identify key metabolites, metabolic pathways, and endometrial microbiota potentially involved in the development of clinically diagnosed AM and EM patients, along with HC, as elucidated by metabolome–microbiome data. Furthermore, we delineate the similarities and differences in the composition of metabolites, microbiota, and gene expression between the AM and EM patients (Fig. 7E). The shared characteristics may offer insights into clinical treatment strategies for individuals affected by both conditions, while the distinctions could provide valuable clues to advance future research into the pathogenesis of these disorders.

A substantial body of evidence underscores the critical role of lipids in all phases of inflammation, emphasizing their interactions with cell surface and intracellular sensors that regulate inflammatory cell signaling and gene expression (64). Dysregulated lipid metabolism is increasingly recognized as a hallmark of various disorders, including AM (32) and EM (65). Our findings reveal that lipids such as PC(40:8), glycerophosphocholine, and sphingosine (d18:2)_RT434 were downregulated in the EM group. This aligns with prior studies (66–69), which have reported decreased lipid levels in patients with EM (70). Importantly, PCs serve as precursors for sphingomyelin and prostaglandins, both of which play crucial roles in mediating the anti-inflammatory processes associated with the pathophysiology of EM (71). Additionally, these lipids contribute to proliferative growth through the enzymatic production of lysoPC (72, 73). In conjunction with the alterations in lipid metabolism, we observed significant changes in purine metabolism among the EM patients. Purines are essential components of DNA and RNA, and their upregulation may correlate with increased biological processes such as cell proliferation and protein synthesis (74). Specifically, xanthine can be biosynthesized from guanine or through the action of purine nucleoside phosphorylase. In the present study, we identified elevated levels of xanthine and guanine in the EM patients, corroborating findings from previous research (75). This suggests that dysregulation of purine metabolism may contribute to the occurrence and progression of EM. Furthermore, some studies indicate that purine metabolites play a role in regulating oocyte meiosis, implying that the observed decline in oocyte quality among EM patients could be linked to the upregulation of purine metabolism (76). We also noted elevated levels of indole metabolites, such as indole and imidazoleacetic acid, likely resulting from tryptophan metabolism. Tryptophan derivatives, including indole, can activate exogenous receptors, such as aryl hydrocarbon receptors, to induce immunological tolerance. Thus, the upregulation of tryptophan and related indole derivatives in EM may aid the ectopic endometrial tissue in evading autoimmune attacks (77).

Current research into the metabolic signatures of AM is limited, leaving a significant gap in our understanding of how metabolic pathways influence this condition (29, 32, 78). Our study found elevated levels of N1-methyl-4-pyridone-3-carboxamide (4PY) in both the AM and EM patients. 4PY, a terminal metabolite of niacin, exhibits biological activity and can trigger vascular inflammation, a process regulated by the NAD synthesis gene *ACMSD* (aminocarboxymuconate semialdehyde decarboxylase). This may contribute to a persistent and chronic inflammatory response (79). Notably, consistent with findings in EM, we observed a downregulation of lipids such as hexadecenedioylcarnitine, 2-hydroxystearic acid, PC(40:8), sph(d18:2)_RT434, and LPE(16:0) in the AM group, indicating similar lipid alterations in both conditions. Additionally, we identified a significantly altered metabolite in AM, 4-aminobutyric acid (GABA), a neuroactive compound involved in the metabolism of several amino acids including β-alanine, alanine, aspartate, glutamate, arginine, and proline. This metabolite is activated through interactions between bacterial products and host receptors, which can influence neuronal transmission, pain perception, inflammation, and hormone release (19, 80). Our findings align with those of Song et al., who reported altered amino acid levels in the myometrium of patients with AM (32). Furthermore, of the 11 altered metabolites observed in AM, two were derived from microbial sources; likewise, four of the 13 altered metabolites in EM were microbial in origin. However, the metabolic pathways associated with the microbiota revealed marked differences between the two conditions. This suggests that the disparities in the metabolic profiles of endometrial samples from the AM and EM patients may be partially attributed to variations in the microbiota present in the respective disease groups.

Intracellular microbiota has attracted considerable attention in recent years, particularly regarding their impact on human cancer (81, 82). In this study, we employed the 5R 16S rRNA sequencing method, which significantly enhances both coverage and resolution, allowing for the identification of endometrial microbiota at the species level (36). To ensure the integrity of our results, we implemented a rigorous contamination removal procedure to mitigate DNA contamination. The alpha diversity levels observed among the AM, EM, and HC groups in our study cohort align with findings reported by other researchers (21, 25–27). Additionally, the significant variations in beta diversity of endometrial microbiota between the AM/EM and HC groups further corroborate earlier studies (21, 25, 27). The abundance of bacteria enriched in the AM group, such as *Prevotella* and *Actinomyces* (21, 27), along with EM-enriched bacteria like *Actinomyces*, *Blautia*, *Escherichia*, *Prevotella*, and *Corynebacterium* (25, 83–86), supports findings documented in previous research. Notably, we observed a depletion of specific microbiota in women with AM or EM, including *Citrobacter* and *Enterobacter*, which had been previously reported to be more abundant (23, 25). Furthermore, the predominant taxa in patients with AM and EM were primarily non-*Lactobacillus* dominant, featuring significant fractions of *Corynebacterium* and *Actinomyces*. These results are consistent with findings from Chen et al. (23), Lin et al. (27), Zhang et al. (87), and Xiao et al. (88) but differ from several other studies that identified *Lactobacillus* as the dominant endometrial microbiota (24, 89, 90). The discrepancies between our findings and those of previous research may be attributed to differences in sample type, sequencing methodologies, analytical techniques, sample collection protocols, and study populations.

Interestingly, *Corynebacterium* sp., *Bacteroides* sp., and *Collinsella* sp., which have not been previously reported, were found to be overabundant in patients with AM or EM in our study. *Corynebacterium*, a common component of both human and mouse skin microbiota, has been shown to interact with the immune system, leading to inflammation (91). Specific strains within this genus have been implicated in the development of nasopharyngeal cancer due to their ability to stimulate intratumoral inflammation by upregulating the CXCL8-CXCR1/2 axis (92). *Bacteroides*, a group of anaerobic, bile-resistant, gram-negative rods, constitutes a significant portion of the human gut microbiota and is crucial for maintaining metabolic homeostasis and immune regulation in their host. They assist in lipid metabolism, which may influence outcomes related to obesity and systemic inflammation (93, 94). For instance, *Bacteroides* species produce pro-inflammatory lipopolysaccharides (LPS) that exacerbate systemic inflammation, a key factor in the pathogenesis of Alzheimer’s disease (95). One notable species, *B. fragilis*, is known to cause colonic inflammation in mice by secreting exotoxins that disrupt epithelial junctions (96). Furthermore, *C. aerofaciens*, recognized as a pathobiont that fosters pro-inflammatory immune responses, has been linked to various conditions such as type 2 diabetes and psoriatic arthritis (97, 98). In models of collagen-induced arthritis, *C. aerofaciens* exacerbates the condition by increasing gut permeability and enhancing the expression of IL-17, CXCL1, and CXCL5 in intestinal epithelial cells (98). Collectively, these findings suggest that these bacterial populations may play a role in the development of AM or EM.

The microbiota plays a critical role in regulating estrogen levels by secreting β-glucuronidase (GUS), an enzyme that facilitates the degradation of estrogen. This process increases the reabsorption of free estrogen, leading to elevated circulating estrogen levels (99). Such an increase may enhance interactions with estrogen receptors ERα and ERβ (100). Various bacterial genera within the gut microbiome, including *Bacteroides*, *Bifidobacterium*, *Escherichia coli*, and *Lactobacillus*, are known to produce GUS (99, 101). Notably, patients with EM have been shown to have a higher prevalence of *E. coli* in their fecal samples (102, 103), supporting the notion that the microbiota can raise circulating estrogen levels, potentially creating a high-estrogen environment that promotes the progression of EM (18). In the present study, we identified three bacterial species associated with AM—*Bacteroides dorei*, *E. cloacae*, and *Prevotella stercorea* that carry the *gus* gene, with two of them exhibiting increased abundance in the AM group. Meanwhile, among the 11 bacterial species carrying the *gus* gene specific to EM, *B. dorei*, *Bacteroides intestinalis*, *Bacteroides ovatus*, *Bacteroides uniformis*, *Bacteroides caccae*, *Bacteroides coprocola*, *Bacteroides plebeius*, *Bacteroides stercoris*, *C. aerofaciens* and *Parabacteroides merdae* showed increased abundance, with the exception of *E. cloacae*. These results suggest that similar pathogenetic mechanisms related to GUS production exist between AM and EM, indicating that further research is needed to elucidate the factors that stimulate GUS production by specific microbiota.

Single-omics biomarkers, such as those derived from the microbiome and metabolome, are increasingly recognized as key factors in understanding the pathogenesis of AM and EM (21, 29, 70, 104). Nonetheless, there remains a significant gap in research that combines both microbiome and metabolome data through multi-omics approaches in the context of AM and EM. In the present study, we identified a cluster of endometrial microbiota and metabolite-based signatures that were either enriched or depleted in individuals with AM and EM, allowing for precise predictions of each phenotype compared to HC. Notably, a core set of metabolic compounds, associated with AM (e*.*g*.*, N-acetylaspartylglutamylglutamate) and EM (e*.*g*.*, indole), exhibited greater predictive accuracy over microbial signatures. Consequently, our findings provide promising evidence for an *in situ*-based differentiation between AM and EM.

Our study demonstrates several notable strengths that warrant attention. Firstly, it represents the first comprehensive investigation into the simultaneous assessment of endometrial microbiota composition and its metabolic profiles within a well-characterized cohort of patients with AM and EM. These unique findings open multiple avenues for new mechanistic research. Secondly, we successfully distinguished between endometrial metabolites produced by the microbiome and those synthesized by the host, while also elucidating the metabolic pathways associated with these identified metabolites. Nevertheless, we must acknowledge certain limitations. Firstly, the normal control endometrial tissue used in our research was obtained from patients undergoing total hysterectomy for uterine leiomyomas, which may not fully represent healthy endometrial tissue. Secondly, we did not include validation cohorts, and all participants were recruited from a single center. Future multicenter studies with geographically diverse cohorts are essential for further validating these findings. Thirdly, our results are largely data-driven and require verification through both *in vivo* and *in vitro* experiments involving specific bacteria and their metabolites. Lastly, while non-targeted metabolomics based on LC-MS provides a comprehensive and unbiased analysis, accurately annotating many metabolites remains a challenge. This limitation may lead to information loss and the potential introduction of interference from exogenous substances.

In conclusion, this cross-sectional study employing multi-omic approaches provides novel insights into the pathogenesis of EM and AM, uncovering both distinct and interconnected metabolic and microbial signatures. Metabolomic and microbiomic analyses revealed unique and shared features, with common pathways such as linoleic acid metabolism suggesting potential therapeutic targets for both diseases, while distinct signatures offer promising diagnostic biomarkers. Integrative analysis with transcriptomic data further elucidated specific pathways related to immune response and signaling transduction, enhancing our understanding of the underlying mechanisms. Machine learning models demonstrated high predictive accuracy in differentiating AM, EM, and HC, highlighting their clinical potential for personalized treatment strategies and refined disease classification. Collectively, these findings not only illuminate potential inconsistencies in pathogenetic mechanisms but also open new avenues for further research and clinical applications, paving the way for more targeted and effective interventions.

## MATERIALS AND METHODS

### Patient cohorts

We recruited 244 women of reproductive age (18 to 50 years) who were undergoing hysterectomy at the Shanghai First Maternity and Infant Hospital between January 2022 and December 2023. The exclusion criteria were as follows: (i) a prior diagnosis of autoimmune, inflammatory, and/or neoplastic diseases; (ii) the use of antibiotics, antimycotics, or barrier contraceptive methods within the preceding 30 days; (iii) pregnancy, lactation, or menstruation at the time of sampling; and (iv) positive for human papillomavirus (HPV), HIV, or hepatitis B/C. Participants were classified into the EM group or the AM group based on lesion identification during hysterectomy, which was further confirmed by pathologists. For the control group, healthy eutopic endometrium (HC) samples were collected from patients without EM or AM who were undergoing hysterectomy for non-submucosal uterine leiomyomas, which help minimize any impact on the collection of endometrial tissue. Among the enrolled women, 91 were classified as EM, 59 as AM, and 97 as HC. All samples were collected in the operating room to minimize contamination, immediately frozen in liquid nitrogen, and stored at −80°C.

### Non-targeted metabolomic profiling

For metabolite extraction, the collected samples were thawed on ice, and metabolites were extracted from each sample (20 ± 5 mg) using pre-cooled extraction solvent (methanol:acetonitrile:water = 2:2:1). The mixture was vortexed and then stored at −20°C overnight. Following centrifugation at 15,000 × *g* for 15 minutes, the supernatant was transferred to a new Eppendorf tube for freeze-drying. The samples were subsequently re-dissolved in 80% methanol containing 5 ppm L-phenylalanine-d8 and stored at −80°C until LC-MS analysis. QC samples were prepared by mixing equal volumes of extraction solvent from 16 randomly selected samples from each group, with the volume of each QC sample corresponding to that of the individual samples.

The analysis of metabolites was conducted using an Orbitrap Fusion mass spectrometer (Thermo Fisher Scientific, USA) in both positive and negative ion modes. Chromatographic separation was achieved with a Thermo Vanquish ultra-high-performance liquid chromatography system (Thermo Fisher Scientific, USA), utilizing an Accucore-150-Amide-HILIC column (100 × 2.1 mm, 2.6 µm, Thermo Fisher Scientific, USA) maintained at 40°C. The mobile phase comprised solvent A (acetonitrile:water = 95:5, with 10 mM ammonium acetate and 0.1% formic acid) and solvent B (acetonitrile:water = 50:50, with 10 mM ammonium acetate and 0.1% formic acid). The gradient elution conditions were as follows: 2% solvent A for 0 to 0.5 minutes; 2%–98% solvent A from 0.5 to 9.0 minutes; 98% solvent A from 9.0 to 11.0 minutes; 98%–2% solvent A from 11.0 to 11.1 minutes; and 2% solvent A from 11.1 to 13.0 minutes. The flow rate was maintained at 0.3 mL/min, and the injection volume was 2 µL. The mass spectrometer parameters were configured as follows: spray voltages were set to +3.4 kV in a positive mode and −2.7 kV in a negative mode, with a transfer tube temperature of 300°C. The sheath gas flow rate was configured to 40 arbitrary units (arb.), the auxiliary gas flow rate was set to 10 arb., the sweep gas flow rate was 1 arb., and the S-lens radio frequency (RF) level was adjusted to 60.0 V. Data acquisition was performed in full MS/data-dependent MS^2^ (dd-MS^2^) mode. The full MS scan range was *m/z* 70–850 at a mass resolving power of 120,000 full width at half maximum (FWHM). The isolation window for the dd-MS^2^ was set to 1.2 *m/z* with a resolution of 15,000 FWHM. Normalized collision energies (NCEs) were calibrated at 15, 30, and 45. Data acquisition and processing were performed using Xcalibur 4.1 software (Thermo Fisher Scientific, USA). Mass accuracy calibration was conducted after every 20 samples to ensure precise measurements. To monitor the stability of the LC-MS system throughout the data acquisition process, a quality control sample was analyzed after every 10 samples.

The data processing protocol adhered to methods outlined in prior publication (37). Initially, the raw MS data files (.raw) were converted to mzXML format using ProteoWizard (v3.0.20196). Subsequently, mzXML data files for the samples were grouped for peak detection, retention time correction, and peak alignment using XCMS (v3.4.1). Following this, missing value imputation and data normalization were performed using MetaboAnalyst (v5.0). Metabolic peaks exhibiting a coefficient of variation (CV) of less than 50% in QC samples were selected for further analysis. Metabolite annotation was conducted using MetDNA (v1.4.1; http://metdna.zhulab.cn/), with separate analyses performed in both positive and negative modes. The selection of differential metabolites was executed using the Mann–Whitney *U* test. PCA was carried out using MetaX to identify specific differences between groups, with criteria for the screening of differential metabolites defined as VIP > 1, adjusted *P* < 0.05, and fold change > 2. *P* values in both PCA and PLS-DA plots were computed by PERMANOVA using Bray–Curtis distance through the R package vegan. Additionally, MetOrigin (http://metorigin.met-bioinformatics.cn/) was employed for the traceability analysis of differential metabolites, utilizing its simple MetOrigin analysis model. This included origin and function analyses, as well as the creation of a Sankey network to link all potential bacteria involved in specific metabolic reactions. This approach aids readers in identifying the significant interactions between bacteria and metabolites.

### High-throughput 5R 16S rRNA gene sequencing

DNA was extracted from frozen samples using the CTAB method with the DP302-02 kit (TianGen, Beijing, China) and was quantified using Qubit (Invitrogen, USA). The amplification and sequencing of the 16S rRNA gene were conducted as previously described with minor modifications (36). A set of bacterial primers specifically designed to target the V5 region of the 16S rRNA gene was employed in the amplification process, with each primer utilized at a concentration of 0.1 µM. The forward primers used were F1 (TGGCGAACGGGTGAGTAA), F2 (ACTCCTACGGGAGGCAGC), F3 (GTGTAGCGGTGRAATGCG), F4 (GGAGCATGTGGWTTAATTCGA), and F5 (GGAGGAAGGTGGGGATGAC). The corresponding reverse primers included R1 (AGACGTGTGCTCTTCCGATCTCCGTGTCTCAGTCCCARTG), R2 (AGACGTGTGCTCTTCCGATCTGTATTACCGCGGCTGCTG), R3 (AGACGTGTGCTCTTCCGATCTCCCGTCAATTCMTTTGAGTT), R4 (AGACGTGTGCTCTTCCGATCTCGTTGCGGGACTTAACCC), and R5 (AGACGTGTGCTCTTCCGATCTAAGGCCCGGGAACGTATT).

The amplification reaction was performed using Phusion Hot Start Flex 2× Master Mix (NEB, #M0536L), incorporating 50 ng of template DNA in a total volume of 25 µL. The amplification protocol included an initial denaturation step at 98°C for 30 seconds, followed by 30 cycles comprising denaturation at 98°C for 10 seconds, annealing at 62°C for 15 seconds, extension at 72°C for 35 seconds, and a final elongation step at 72°C for 5 minutes. To incorporate barcodes and Illumina adapters into the amplicons, a secondary PCR was performed using five forward primers (0.1 µM each). The forward primers utilized were as follows: FF1-AATGATACGGCGACCACCGAGATCTANNNNNNNNTACACTCTTTCCCTACACGACGCTCTTCCGATCTTGGCGAACGGGTGAGTAA, FF2-AATGATACGGCGACCACCGAGATCTANNNNNNNNTACACTCTTTCCCTACACGACGCTCTTCCGATCTACTCCTACGGGAGGCAGC, FF3-AATGATACGGCGACCACCGAGATCTANNNNNNNNTACACTCTTTCCCTACACGACGCTCTTCCGATCTGTGTAGCGGTGRAATGCG, FF4-AATGATACGGCGACCACCGAGATCTANNNNNNNNTACACTCTTTCCCTACACGACGCTCTTCCGATCTGGAGCATGTGGWTTAATTCGA, FF5-AATGATACGGCGACCACCGAGATCTANNNNNNNNTACACTCTTTCCCTACACGACGCTCTTCCGATCTGGAGGAAGGTGGGGATGAC. Additionally, one reverse primer (0.1 µM, RR5-CAAGCAGAAGACGGCATACGAGATNNNNNNNNGTGACTGGAGTTCAGACGTGTGCTCTTCCGATCT) was employed, which contained an eight-nucleotide barcode. To enhance sequencing throughput and minimize index contamination, unique indices (TA) were included at both the P5 and P7 ends. A 50 ng aliquot of the amplicon underwent further amplification in a 25 µL reaction volume, consisting of six cycles involving denaturation at 98°C for 10 seconds, annealing at 64°C for 15 seconds, and extension at 72°C for 25 seconds. The resulting PCR products were verified via 2% agarose gel electrophoresis. During the DNA extraction process, ultrapure water served as a negative control instead of a sample solution to mitigate the risk of false-positive PCR results. The PCR products were subsequently purified using AMPure XT beads (#A63880, Beckman Coulter, Danvers, MA, USA) and were quantified with Qubit. The amplicon pools were prepared for sequencing, with both the size and quantity of the amplicon library assessed using an Agilent 2100 Bioanalyzer (Agilent, USA) along with the Library Quantification Kit for Illumina (#KK4844, Kapa Biosciences, Woburn, MA, USA). Finally, the libraries were sequenced on a NovaSeq 6000 system using a paired-end 2 × 150 protocol (LC Bio Technology Co., Ltd., Hangzhou, China).

For data quality control, the raw data undergo sample data splitting, and the statistical analysis of sequencing volume and high-quality base ratio for each sample is conducted based on barcode information. To ensure the reliability of subsequent analyses, we meticulously screen and filter the raw sequence data using the following steps: (i) Cutadapt (v1.9) is employed to identify potential 3′ end adapter sequences, followed by truncation at the identified adapter sequences. (ii) After the removal of the 3′ end adapter sequences, fqtrim (v0.94) is utilized to perform quality screening on the sequences via a sliding window method. This method involves scanning a window size of 10 bp, starting from the first base position at the 5′ end, and requiring that the average base quality within the window be ≥Q20 (indicating a sequencing accuracy of bases >99%). Sequences are truncated at the 3′ end at the first window where the average quality falls below Q20. (iii) Sequences shorter than 100 bp after truncation are discarded. (iv) Additionally, sequences with a content of ambiguous bases (N) exceeding 5% after truncation are removed. Following these processing steps, a set of effective sequences (Clean Data) suitable for subsequent analysis is generated, and the proportion of these sequences relative to the original sequencing data is statistically calculated (Clean%).

For species annotation and the calculation of relative abundance, we employed the short multiple regions framework (SMURF) (105) analysis pipeline to integrate quality-filtered sequence data for preliminary identification of the microbial communities in the samples and to assess their relative abundances. SMURF utilizes the expectation-maximization algorithm to integrate and reconstruct sequences from multiple short amplification regions, referencing the Greengenes database (May 2013 version) established by Nejman et al. (36) to identify the most probable 16S sequence sets for bacterial taxonomic identification and relative abundance assessment. To mitigate the influence of low-abundance noise on subsequent analyses, we normalized the sequence read counts for each sample, discarding samples with a total read count of less than 1,000 (including negative controls) and bacterial data with a relative abundance of less than 10^−4^. Subsequently, we applied a stringent contaminant filtering process to eliminate common environmental and experimental contaminants (36), setting a threshold of 30% prevalence to identify potential contaminants. The microbial information remaining after the removal of these contaminants was deemed representative of the microbial communities present in the tissues.

For the diversity analysis, we employed QIIME 1 (v1.8.0) to conduct both alpha and beta diversity assessments based on the microbial community abundance table, followed by visualization using the vegan package (v2.6.2). Alpha diversity was primarily evaluated using indices such as Chao1, observed species, Shannon, and Simpson, which together reflect the richness and evenness of the microbial communities. These indices were calculated using the Kruskal–Wallis rank-sum test and the Mann–Whitney *U* test. Beta diversity was determined through PERMANOVA based on Bray–Curtis distance, and the results were visually represented using PCoA to observe the differences in microbial community composition among groups. Furthermore, the distribution of shared and unique microbiota at the species level was visualized using a Venn diagram, generated with the Venn Diagram package (v1.7.3).

To assess the composition and distribution of the most abundant species across various taxonomic levels, as well as to compare microbial compositions among different groups, we utilized the ggplot2 package (v3.4.0) to calculate the relative abundance of each bacterial species at each taxonomic level in relation to the total species abundance. Stacked bar charts were constructed to visually represent the distribution of bacterial species across different groups at various taxonomic levels. For the inter-group microbial community differential analysis, we generated a heatmap using the Heatmaps package (v1.0.12). A Mann–Whitney *U* test was conducted for comparative analysis, with a significance threshold established at *P* < 0.05. The random forest model utilized differential metabolic and microbial species as predictors. This model, which consisted of 500 decision trees, identified significant variables for prediction employing random forest software (v4.7-1.1). A total of 20 trials involving 10-fold cross-validation were conducted. The cross-validation error curves generated from these trials, using the graphics package (v4.1.3), were averaged. The minimum error from the averaged curve, along with the standard deviation at that specific point, was designated as the cutoff for acceptable error. Additionally, ROC analysis was performed using the pROC package (v1.18.5), with plots generated against the number of variables.

### Meta-analysis of publicly available matching transcriptomic data sets

The transcriptome data sets utilized in this study were obtained from the Gene Expression Omnibus (GEO) public database at the National Center for Biotechnology Information (NCBI) using the search term “adenomyosis/endometriosis.” The criteria for data set inclusion in the meta-analysis were as follows: (i) the data set must consist of genome-wide mRNA expression sequencing data and (ii) each data set must contain a minimum of three samples per group. Ultimately, nine data sets in FASTQ format met these criteria for inclusion: GSE135485, GSE153739, GSE153740, GSE157718, GSE171653, GSE185392, GSE193928, GSE212787, and GSE228005.

Quality control for each FASTQ file was performed using FastQC (v0.12.1), followed by the removal of low-quality sequences and the trimming of adapter sequences using Trim Galore (v0.6.10). The processed sequencing data were then aligned to the *Homo sapiens* GRCh38 reference genome using HiSat2 (v2.2.1), with read counts generated via featureCounts (v2.0.3), yielding the corresponding expression matrix. The nine data sets comprised a total of 166 samples, which included 74 samples classified as EM, 31 as AM, and 61 as HC. Due to the diverse sources of the samples, we performed quality control at both the gene and sample levels. At the gene level, we retained genes expressed in at least 75% of the samples, and at the sample level, we retained samples with a total read count of no fewer than 10 million. Following these quality control measures, 93 samples were included in the final analysis, consisting of 19 EM samples, 22 AM samples, and 52 HC samples.

Subsequently, we applied the Combat-Seq package (106) to perform batch correction on the 93 samples, resulting in a final expression matrix. Two matrices were constructed: one for EM samples and another for AM samples compared to the HC. DEGs were identified using DESeq2 (v1.22.2), applying a threshold of |log_2_FC| ≥ 0.585 and a *P* value < 0.05. Finally, functional enrichment analysis of the DEGs was performed using the open-access KEGG database (v111.0), employing a hypergeometric test with a significance threshold of 0.05, and the resulting *P* values were adjusted using the FDR method to control for multiple testing. Data analysis was primarily conducted using the R software (v4.3.0).

### Quantitative real-time polymerase chain reaction (qRT-PCR) analysis

Total RNA was isolated from endometrial tissues using TRIzol reagent (Invitrogen Cat #15596026) and subsequently reversed transcribed into cDNA utilizing 1 µg of total RNA with the PrimeScript RT Reagent Kit with gDNA Eraser (RR047A; Takara, Japan) in conjunction with the ABI 7500 Real-Time PCR system (Applied Biosystems, Foster City, CA, USA). qRT-PCR was conducted using the TB Green Premix Ex Taq II (Tli RNaseH Plus) kit (RR820A; Takara, Japan), adhering strictly to the manufacturer’s instructions. The primer sequences utilized for qRT-PCR are detailed in Table S9. Relative expression levels of RNA, normalized to GAPDH, were calculated by assessing the changes in expression using the equation 2^-ΔΔCt^.

### Statistical analysis

Classified data were reported as counts or as means ± standard deviation (SD). Statistical analyses were conducted using the chi-square test for categorical variables and the two-sided unpaired *t*-test for continuous variables. For comparisons between two groups, either the two-sided unpaired *t*-test or the nonparametric Mann–Whitney *U* test was employed, as appropriate. The Kruskal–Wallis rank-sum test and PERMANOVA were utilized for data with non-normal distributions among three groups, when suitable. To construct the metabolic and microbial correlation heatmap, Pearson correlations were calculated using the OmicStudio tools at https://www.omicstudio.cn/tool/62. Additionally, correlations between microbiota and clinical parameters were determined utilizing Pearson correlations, with a significance threshold set at *P* < 0.05. GraphPad Prism 7.0 (GraphPad Software, California, USA) and SPSS software (standard v19.0; IBM) were used for statistical analysis. *P* values are presented as follows: *P < 0.05, **P < 0.01, and ***P < 0.001.

## Data Availability

The 5R 16S rRNA sequencing data reported in this paper have been deposited in GSA with accession number CRA018483. The non-targeted metabolomics data reported in this paper have been deposited in OMIX with accession number OMIX007190. The authors declare that all the data supporting the findings of this study are available within the paper or from the corresponding authors upon request.

## AFTER EPUB

*[This article was published on 22 April 2025 with errors in the author affiliations and Fig. 1. The errors were corrected in the current version, posted on 28 April 2025.]*

## SUPPLEMENTAL MATERIAL

Supplemental Figures msystems.00202-25-s0001.pdf Figures S1 to S6.

Table S1 msystems.00202-25-s0002.xlsx Baseline clinical characteristics of the women included.

Table S2 msystems.00202-25-s0003.xlsx Metabolic peaks.

Table S3 msystems.00202-25-s0004.xlsx MetOriginmetabolite traceability analysis.

Table S4 msystems.00202-25-s0005.xlsx Bacterial species identified using 5R 16S rRNA sequencing.

Table S5 msystems.00202-25-s0006.xlsx Species associated with experimental contamination in DNA extraction and PCR control.

Table S6 msystems.00202-25-s0007.xlsx Species associated with contamination in blank control.

Table S7 msystems.00202-25-s0008.xlsx Enriched KEGG pathways.

Table S8 msystems.00202-25-s0009.xlsx Shared KEGG pathways.

Table S9 msystems.00202-25-s0010.xlsx Primers for qPCR.

ASM does not own the copyrights to Supplemental Material that may be linked to, or accessed through, an article. The authors have granted ASM a non-exclusive, world-wide license to publish the Supplemental Material files. Please contact the corresponding author directly for reuse.
