# Supplementary material for: The inconsistent pathogenesis of endometriosis and adenomyosis: insights from endometrial metabolome and microbiome
Source: mSystems. 2025 Apr 22;10(5):e00202-25. doi: 10.1128/msystems.00202-25 (PMC12090731; doi:10.1128/msystems.00202-25)
Supplement: Supplemental Figures — Figures S1 to S6. [file msystems.00202-25-s0001.pdf]

Supplemental information

Integrated analysis of endometrial metabolome and microbiome reveals inconsistent associations between adenomyosis and endometriosis

Chao Li[1,2,‡], Xinxin Xu[3,‡], Xiaojie Zhao[1], Bin Du[1,*]

[1]Department of Pathology, Shanghai First Maternity and Infant Hospital, School of Medicine, Tongji University, Shanghai 201204, China

[2]Shanghai Key Laboratory of Maternal Fetal Medicine, Shanghai Institute of Maternal-Fetal Medicine and Gynecologic Oncology, Clinical and Translational Research Center, Shanghai First Maternity and Infant Hospital, School of Medicine, Tongji University, Shanghai 201204, China

[3]Department of Gynecology, Shanghai First Maternity and Infant Hospital, School of Medicine, Tongji University, Shanghai 201204, China

[‡]These authors contributed equally to this work.

[*]For correspondence: Bin Du, dubin@tongji.edu.cn.

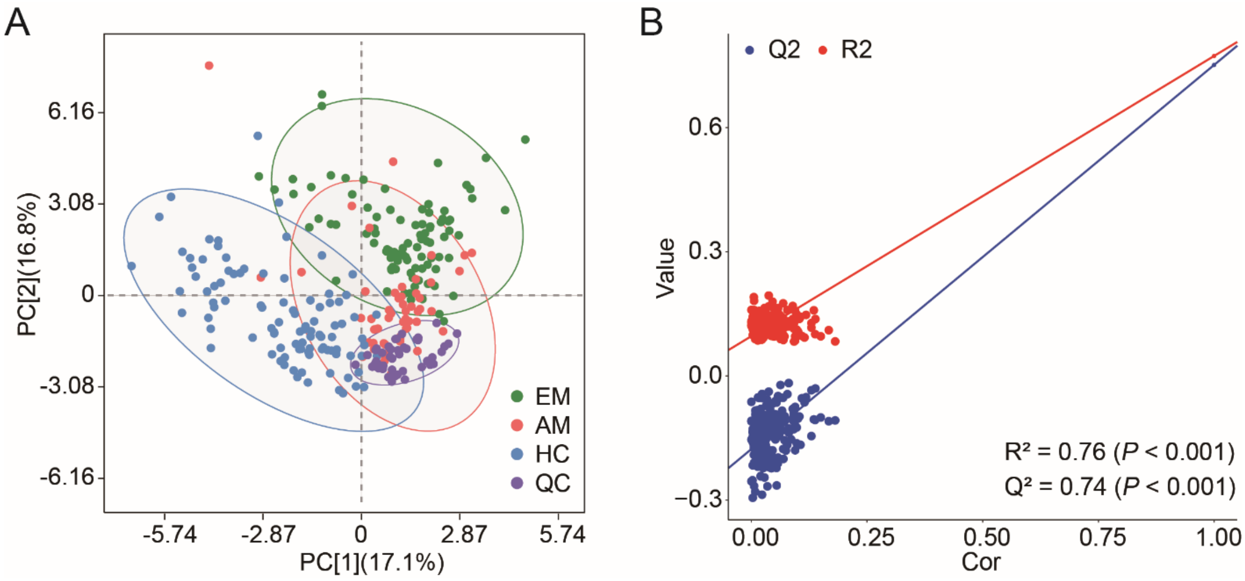

Figure S1. (A) Partial Least Squares Discrimination Analysis (PLS-DA) was conducted for the EM, AM, HC, and quality control (QC) samples. (B) The PLS-DA model was validated using a 200-time permutation test combined with 7-fold cross-validation.

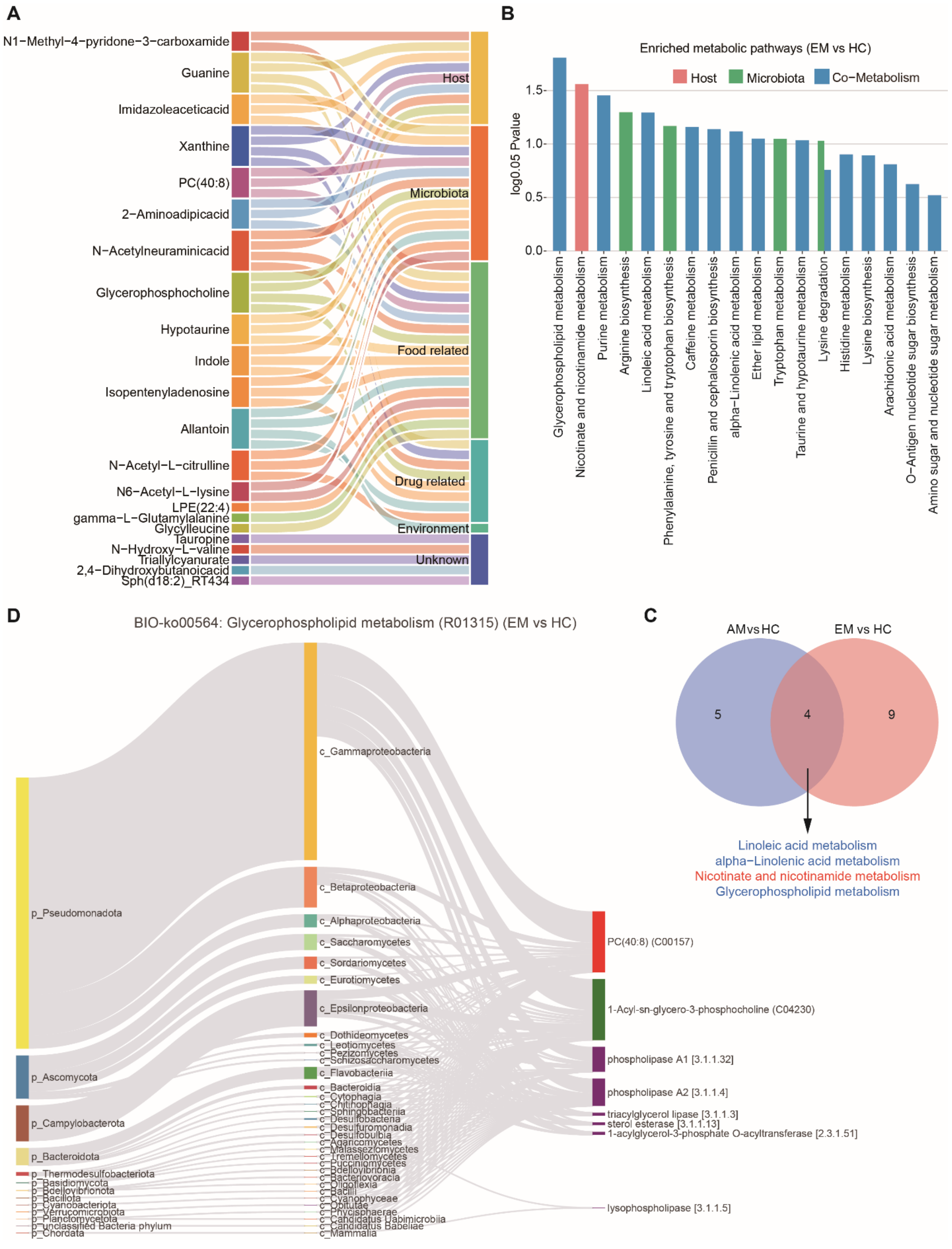

Figure S2. Metorigin metabolite traceability analysis and Sankey diagram of differential

metabolites. (A) Sankey diagram illustrates the origins of differential metabolites. All the thirteen microbial-associated metabolites and nine host-associated metabolites were originated from multiple sources (including host, microbial food, drugs, or environmental factors), suggesting a degree of uncertainty in the annotation of these metabolites. (B) Histogram depicts the results of the enrichment analysis for differential metabolic pathways. (C) Venn diagram illustrates the shared cometabolic pathways between host and bacteria in AM and EM interactions. (D) Sankey diagram detailing the Metorigin analysis of glycerophospholipid metabolism. Dark red bars, metabolic substrates; dark green bars: metabolic products, purple bars: metabolic enzymes.

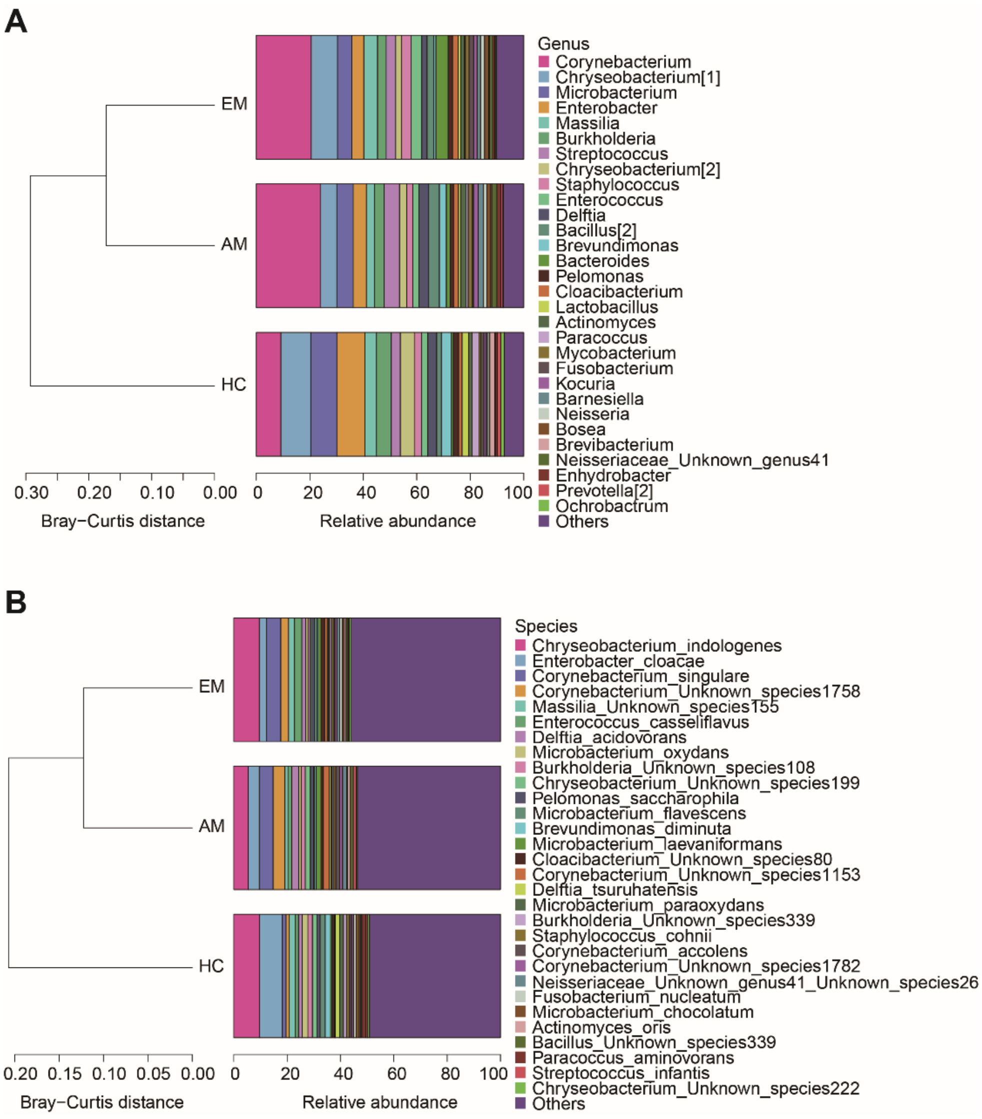

Figure S3. Hierarchical clustering using Bray-Curtis distance based on the average relative proportions of the top 30 most abundant genera (A) and species (B) across the three groups examined. Ug, unknown genus; Us, unknown species.

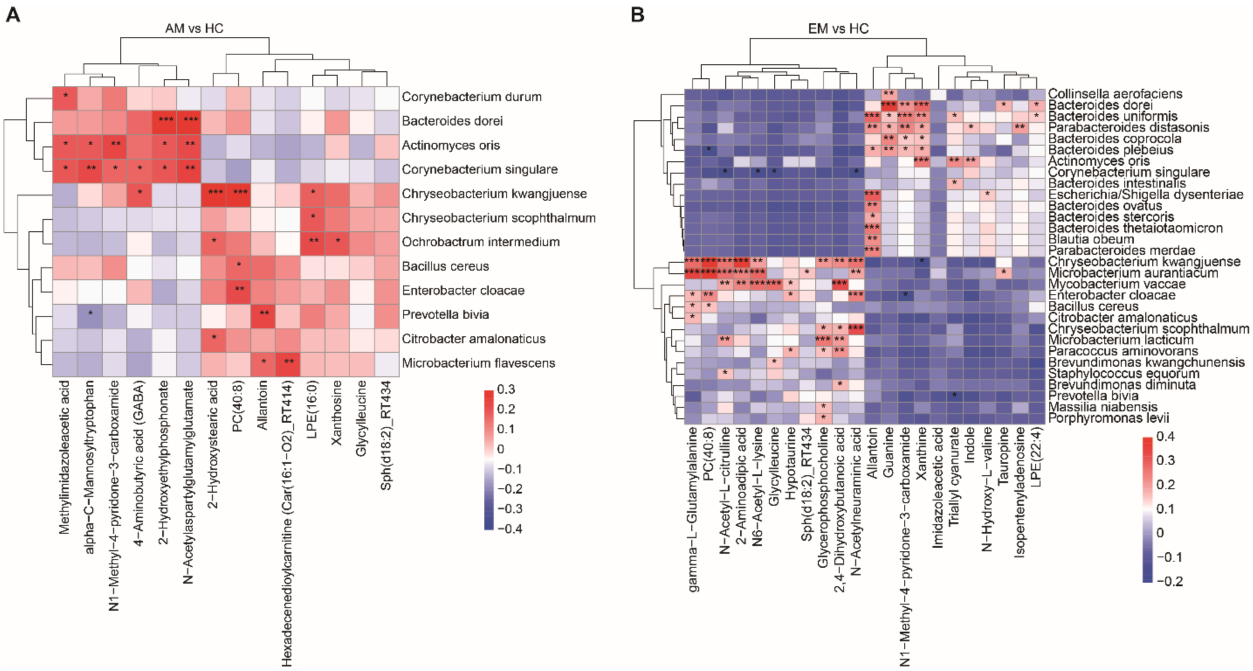

Figure S4. Heatmap illustrates the Pearson correlation coefficients, highlighting the differences in the abundances of species and metabolites between patients with AM and HC (A), as well as from patients with EM and HC (B). Only species that exhibited a correlation with at least one metabolite at a significance level of $p < 0.05$ (*), $p < 0.01$ (**), or $p < 0.001$ (***) are presented.

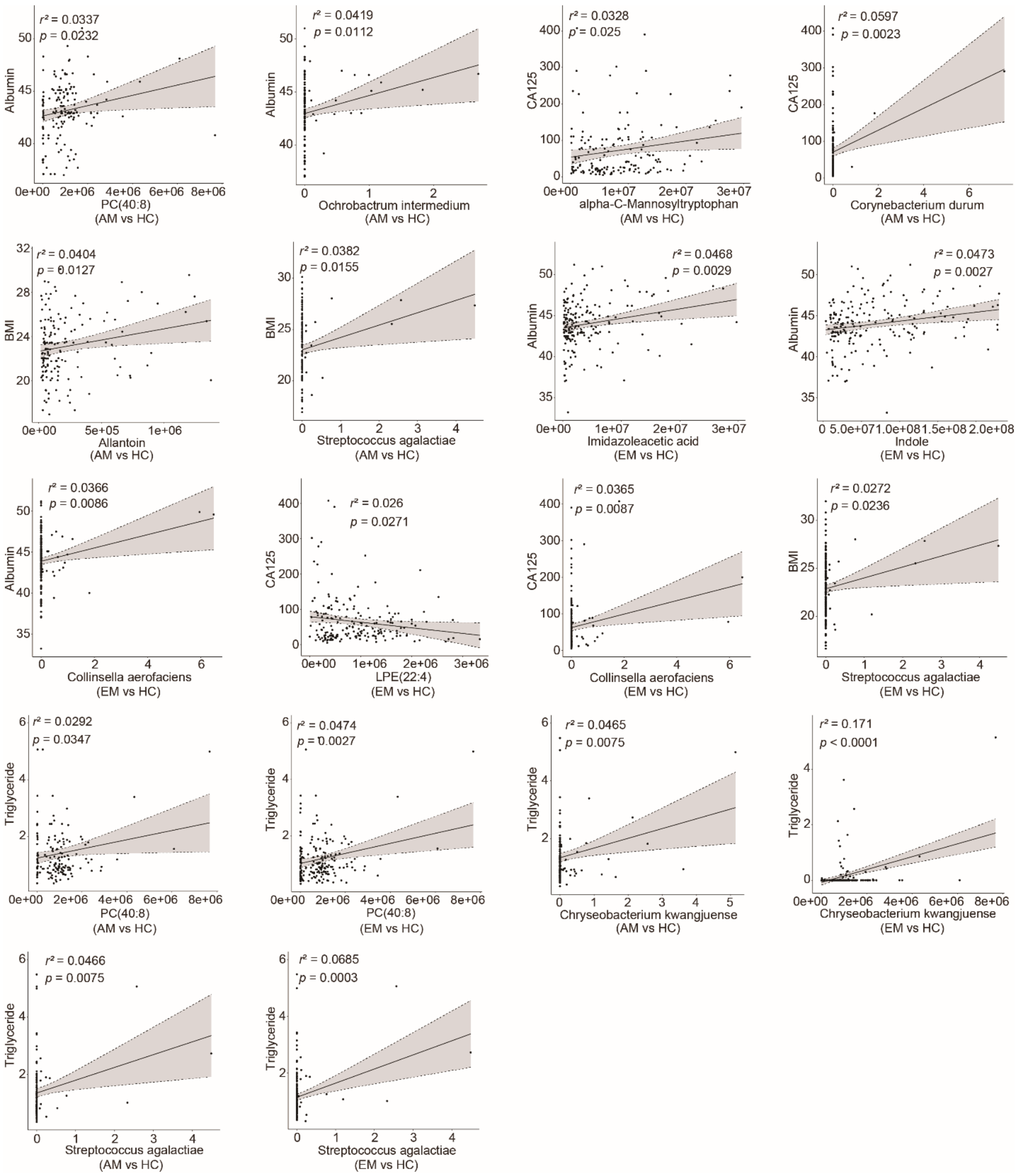

Figure S5. Linear regression analyses between specific differential metabolites or microbiota and clinical indices in the two comparison groups. The solid black line indicates a statistically significant linear association ($P < 0.05$), with shaded areas representing 95% confidence intervals.

Each black dot represents an individual sample.

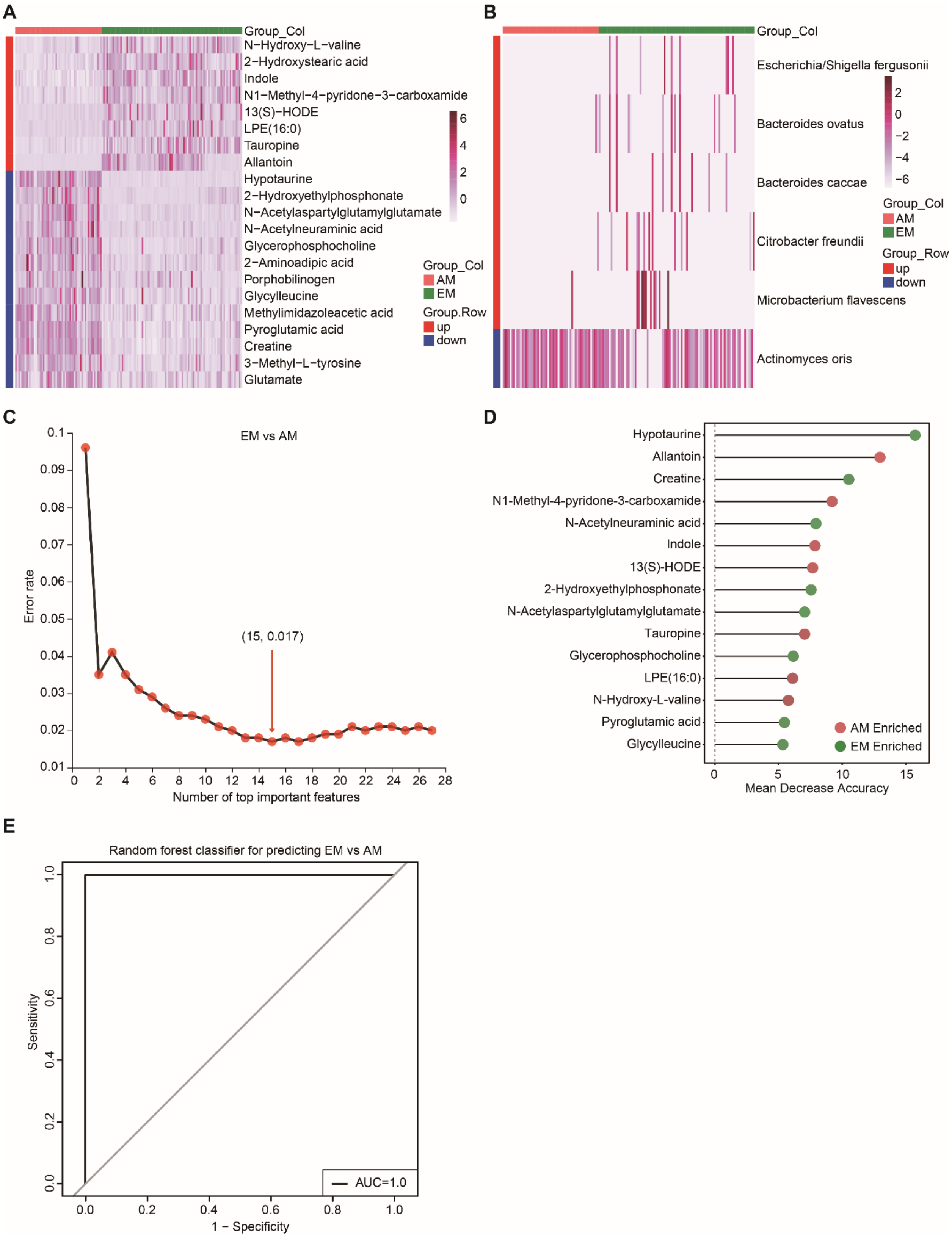

Figure S6. Multi-omics signatures-based predication between AM and EM. (A) Heatmap

displays the significantly altered metabolites between AM and EM groups. (B) Heatmap illustrating the differentially abundant bacterial species between the AM and EM groups. (C) The cross-validation curve illustrates the results from 20 trials of 10-fold cross-validation process comparing the EM group with the AM group, based on the differential metabolic and microbial features. The number of features corresponding to the minimum cross-validation error is highlighted in red font. (D) A total of 15 endometrial metabolic biomarkers were identified as achieving the lowest classification error. These biomarkers were obtained using the mean decrease accuracy tool from the random forests (RF) algorithm and were ranked according to their contributions to classification accuracy following permutation analysis. The color associated with each biomarker indicates its enrichment in the AM (red), or EM (green) participants. (E) The receiver operating characteristic (ROC) curves of the RF model, based on discriminatory signatures, are presented for the 147 samples in this cohort.
